# A novel mechanism for A-to-I RNA-edited CYP1A1 in promoting cancer progression in NSCLC

**DOI:** 10.1186/s11658-025-00718-6

**Published:** 2025-04-02

**Authors:** Zhipeng Wang, Yan Wu, Ziqi Ding, Xinru Xiao, Yanhua Huang, Zhiguang Liu, Qian Zhang

**Affiliations:** 1 Department of Respiratory and Critical Care Medicine, the Second People’s Hospital of Changzhou, the Third Affiliated Hospital of Nanjing Medical University, Changzhou, 213164 China; 2 Changzhou Medical Center, Nanjing Medical University, Changzhou, 213164 China; 3 State Key Laboratory of Microbial Metabolism, Joint International Research Laboratory of Metabolic and Developmental Sciences, School of Life Sciences and Biotechnology, Shanghai Jiao Tong University, Shanghai, 200240 China

**Keywords:** A-to-I RNA editing, CYP1A1 gene, Non-small-cell lung cancer, Heme oxygenase-1

## Abstract

**Background:**

Lung cancer is the most frequently diagnosed malignancy and the leading cause of cancer-related mortality worldwide. Similar to other solid tumors, the development of non-small cell lung cancer (NSCLC) is believed to be a multistep process involving the accumulation of genetic and epigenetic alterations. A-to-I RNA editing is a widespread posttranscriptional epigenetic modification that confers specific nucleotide changes in selected RNA transcripts and plays a critical role in the pathogenesis of many human cancers. However, the mechanisms underlying A-to-I RNA editing that act as a potential driver in the pathogenesis of NSCLC progression remain incompletely elucidated.

**Methods:**

Sanger sequencing was performed to validate the CYP1A1_I462V RNA editing event in NSCLC patients. In vitro and in vivo experiments were used to assess the effects of an ADAR1-regulated CYP1A1 and its editing on NSCLC cell growth and metastasis. The crosstalk between CYP1A1_I462V RNA editing and PI3K-AKT signaling was analyzed using RNA sequencing and molecular methods. The functional role of CYP1A1_I462V in the response to oxidative stress was verified through proteomics analysis, co-IP assay, and immunofluorescence assay.

**Results:**

Sanger sequencing analysis identified an increased A-to-I RNA editing ratio of CYP1A1 in NSCLC specimens. This specific RNA editing, regulated by ADAR1, resulted in gain-of-function phenotypes characterized by enhanced tumor progression and more aggressive behavior. The edited form induced the expression of heme oxygenase-1 (HO-1) via PI3K/Akt-dependent activation compared with the wild-type CYP1A1, which led to an enhanced interaction with CYP1A1, thereby promoting the translocation of abundant HO-1 into the nucleus to resist oxidant stress in NSCLC cells.

**Conclusions:**

Our findings highlight that the I462V A-to-I RNA editing event of CYP1A1 drives pulmonary carcinogenesis through inhibiting oxidative stress and suggest that the CYP1A1-HO-1-PI3K/Akt axis may be a potential therapeutic target for NSCLC.

**Graphical Abstract:**

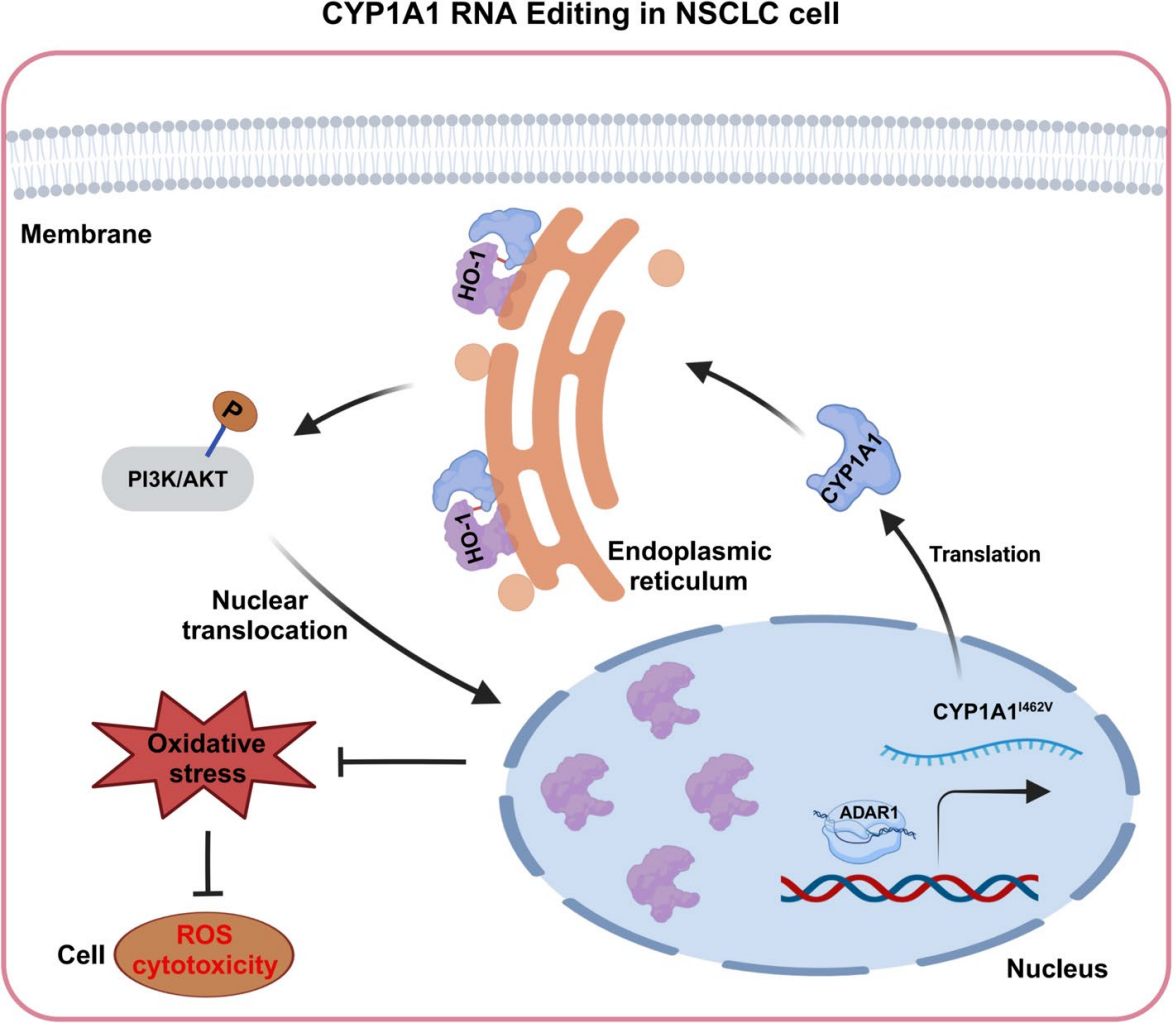

**Supplementary Information:**

The online version contains supplementary material available at 10.1186/s11658-025-00718-6.

## Background

RNA editing is a prevalent posttranscriptional mechanism that specifically modifies RNA nucleotides during the pathological progression of human diseases [1]. The most widespread RNA editing event is adenosine to inosine (A-to-I) mediated by double-stranded RNA-specific adenosine deaminase (ADAR) enzymes in which I is interpreted as guanosine (G) by the translational machinery [2]. Hyperediting represents the most prominent feature among A-to-I editing forms, resulting in an accumulation of edited adenosines within introns and untranslated regions (UTRs) [3, 4]. Abnormalities in A-to-I RNA editing can potentially act as novel drivers for cancer development and progression. For instance, in human hepatocellular carcinoma (HCC), there is an increased frequency of AZIN1 editing at residue 367 (Ser to Gly), leading to inhibition of antizyme tumor-suppressor function and contributing to cancer initiation and progression [5]. Additionally, A-to-I RNA editing events alter the motif involved in binding to the SH2 domain of BLCAP, thereby driving cervical carcinogenesis through the regulation of STAT3 signaling pathway [6]. In view of its importance, A-to-I editing events are considered as an adaptive change of living organisms.

Lung cancer is the most prevalent and lethal malignancy worldwide, accounting for an estimated 1.8 million deaths annually. Among its histological subtypes, non-small-cell lung cancer (NSCLC) is the most commonly encountered [7]. Similar to other human cancers, NSCLC pathogenesis is primarily driven by the accumulation of somatic mutations or epigenetic alterations. A previous study demonstrated that A-to-I RNA editing at position S367G in AZIN1 may confer a gain-of-function effect, promoting NSCLC tumorigenesis through inhibition of antizyme-1-mediated degradation of ornithine decarboxylase (ODC) and cyclin D1 (CCND1) oncoproteins [8]. However, the precise pathological mechanism underlying A-to-I RNA editing in NSCLC remains poorly understood.

Genetic polymorphisms of cytochrome p450 1A1 (CYP1A1) are thought to modulate the risk in lung cancer. In 1990, Kawajiri et al. initially reported an association between CYP1A1 polymorphisms and lung cancer in an Asian study population [9]. Cytochrome P-450s (CYPs) are enzymes involved in the oxidation of various substances. CYP1A1 encodes the primary enzyme responsible for metabolizing polycyclic aromatic hydrocarbons, which may generate highly reactive mutagenic metabolites [10]. Two functionally important single-nucleotide polymorphisms (SNPs) have been identified in the CYP1A1 gene with regard to cancer susceptibility: a T to C base substitution in the 3′-UTR and an A to G transition resulting in a valine-to-isoleucine substitution at codon 462 (I462V) [10, 11]. RNA editing leading to I462V alteration in CYP1A1 is a high-frequent molecular event observed in NSCLC, significantly promoting the malignant phenotypes of cancer cells [8]. However, further investigation is required to elucidate the mechanism underlying epigenetic alterations associated with CYP1A1_I462V editing and NSCLC progression.

In this study, we conducted Sanger sequence analysis on serum samples obtained from patients with NSCLC, as well as primary NSCLC tissues and their respective matched nontumor tissue counterparts. Our findings confirmed the involvement of the CYP1A1_I462V editing event in NSCLC progression. We further demonstrated that ADAR1-mediated edited CYP1A1 induced a stronger PI3K-Akt signaling pathway compared with wild-type CYP1A1. The edited CYP1A1 enhanced the interaction with heme oxygenase-1 (HO-1) and mediated the nuclear translocation of HO-1 to confer resistance to oxidant stress, which promoted cell proliferation and tumor progression in NSCLC. This research reveals a novel mechanism underlying the function of CYP1A1 as a tumor enhancer and provides additional evidence linking I462V A-to-I RNA editing events to tumor development.

## Materials and methods

### Clinical samples

A total of 34 paired human NSCLC and normal adjacent tissues, which were surgically excised and rapidly frozen in liquid nitrogen, were obtained from the Affiliated Changzhou Second People’s Hospital of Nanjing Medical University between 2021 and 2024, accompanied by their corresponding clinicopathological summaries. In addition, a total of 103 serum samples from NSCLC patients and 87 serum samples from healthy subjects were collected from the same hospital. None of these patients underwent preoperative chemotherapy or radiotherapy. This study was conducted in full compliance with the ethical principles of the Declaration of Helsinki and principles of good clinical practices, with approvals from the Ethics Committee of the Affiliated Changzhou Second People’s Hospital of Nanjing Medical University (approval no. KY325-01). Written informed consents were obtained from all participants.

### Cell lines

In this study, human LC cell lines A549 (cat. SCSP-503), H1299 (cat. SCSP-589), and H460 (cat. SCSP-584) and human embryonic kidney 293T cell line (cat. SCSP-502) were obtained from and have been authenticated by the Cell Bank of the Chinese Academy of Sciences (Shanghai, China). The human LC cell lines 95C cell line (cat. SNL-168) and 95D cell line (cat. SNL-253) were purchased from and have been authenticated by SUNNCELL Co., Ltd (Wuhan, China). All cell lines were incubated at 37 ℃ with 5% CO_2_ and cultured in RPMI 1640 medium (Gibco, South America) supplemented with 10% fetal bovine serum (FBS; Gibco, South America).

### RNA extraction, PCR amplification, and qRT-PCR

Total RNA was extracted from tissue samples, serum samples, or cells using TRIzol reagent (Invitrogen, Carlsbad, CA, USA), according to the manufacturer’s standard protocol. Subsequently, 1 μg of total RNA from each sample was reverse transcribed to cDNA using the PrimeScript RT reagent kit (Takara, Tokyo, Japan). The CYP1A1 transcript in the cDNA samples was sequenced utilizing the CYP1A1 F/R primers. The sequencing chromatograms were analyzed with Chromas Lite software (Technelysium, Brisbane, Australia), and the frequency of editing was estimated by ratiometric A/G measurement. Each cDNA sample underwent amplification using HiScript Q RT SuperMix (Vazyme, Jiangsu, China) on an ABI Prism 7900 Sequence detection system (Applied Biosystems, Canada). Glyceraldehyde 3-phosphate dehydrogenase (GAPDH) served as internal standard controls. Finally, the relative RNA expression levels were estimated by the 2^–ΔΔCT^ method. Details of the primers used in this study are described in Table S4.

### CCK-8 assay, EdU assay, and Transwell assay

For CCK-8 assay, A549 cells (3 × 10^3^) or H1299 cells (2.5 × 10^3^) were seeded into 96-well plates (Corning, MA, USA) and cultured for 24, 48, and 72 h. After that, 10 μl of CCK8 (Sangon Biotech, Shanghai, China) solution was added to each well at the appointed time. After 1 h of incubation at 37 ℃, the absorbance at 450 nm was measured using microplate reader (Bio-Rad, USA). The EdU assay was performed utilizing the Cell-Light EdU DNA Cell Proliferation Kit (Sangon Biotech, Shanghai, China) according to the manufacturer’s instructions. After incubation with a concentration of 50 μM EdU for a duration of 2 h, images were captured using fluorescence microscopy (Olympus, Tokyo, Japan). Transwell invasion and migration assays were conducted in 24-well plates, using a 6.5-mm-diameter Transwell chamber with 8-μm pore polycarbonate membrane insert (Corning, MA, USA). After 48 h of transfection, A549 cells (4 × 10^4^) or H1299 cells (3.5 × 10^4^) were plated on the upper chambers coated with or without 50 μl of Matrigel in serum-free medium. RPMI 1640 containing 10% FBS was added to the lower chambers as a chemoattractant, and images were obtained with microscopy. The assay was repeated three times in duplicate. The numbers of cells counted in three random fields were averaged.

### Plasmid construction, shRNA transfection, and lentiviral transduction

The plasmid pcDNA3.1-CYP1A1-WT was designed and synthesized by Hanbio Biotechnology (Shanghai, China). For site-directed mutagenesis, the plasmid pcDNA3.1-CYP1A1-edited was modified with the Fast Mutagenesis System (Transgen Biotech, Beijing, China) to obtain clones containing point mutations. The sequences of shRNAs are described in Table S2, and the primers used in the study are described in Table S3. To generate lentiviral plasmids expressing shRNA, they were cotransfected with pVSV-G and pCMVd8.9 into 293T cells. Viral-containing media were collected, filtered, and concentrated by ultracentrifugation. Stable cell lines with knocked down ADAR1 or ADAR2 expression were obtained by selection with puromycin. The purified PCR products of ADAR1 or ADAR2 were ligated to the pcDNA3.1 vector according to the manufacturer’s instructions. Either the ADAR1 or ADAR2 expression construct was transfected into A549 cells using Lipofectamine 3000 (Invitrogen, Carlsbad, CA, USA). In all transfections, empty pcDNA3.1 served as the control vector. Thirty-six hours after transfection, the transfected cells were harvested for subsequent experiments.

### Animal study

For the NSCLC subcutaneous model, male Balb/c-nu mice aged 6–8 weeks were intravenously injected with FFluc-A549 and FFluc-A549^I462V^ (1 × 10^6^) tumor cells. Tumor formation was monitored in these mice over a period of 4 weeks, and the tumor volume was calculated weekly using the formula volume = 0.5 × length × width × width [12]. For the NSCLC orthotopic model, a sterile incision parallel to the rib cage between ribs 10 and 11 was made to visualize the lung through intact thoracic pleura. Approximately 1 × 10^6^ cells were directly injected into the lung parenchyma at the left lateral dorsal axillary line. Tumor growth was monitored using bioluminescence imaging (BLI) with the AMI system (Ami-HT model, Spectral Instruments Imaging). All animal experiments were conducted according to the rule approved by the Animal Ethics Committee of Yanxuan Biotechnology (Hangzhou) Co., Ltd. (approval no. YXSW2312283817).

### CRISPR/Cas9 system-mediated single-nucleotide mutation

The design of a 20-nt single-guide RNA (sgRNA) was based on the genomic sequences of CYP1A1 (gene ID: 1543) and its potential off-target activity was assessed using the CRISPR Design tool available at http://crispr.mit.edu/. The sequence of sgRNA is described in Table S3. Commercial synthesis of sgRNA oligonucleotides was performed by Sangon Biotech, Shanghai, China. Subsequently, they were subcloned into the BbsI site of the pSpCas9 (BB)-2A-Puro (px459) vector (Addgene plasmid #62988), following the manufacturer’s protocol [13]. Positive transformants were selected using 0.7 μg/mL puromycin, and further experiments involved isolating single cell clones for verification of A549^I462V^ through Sanger sequencing.

### RNA sequencing and analysis

The poly(A) mRNA was isolated from total RNA of A549 and A549^I462V^ using beads containing oligo(dT). Subsequently, short double-stranded cDNA fragments were purified using a QIAquick PCR extraction kit (Qiagen, Germany) and eluted with EB buffer for end repair and addition of an “A” base. The amplified library was sequenced on an Illumina HiSeq™ 2000 sequencing machine (Illumina, USA). Differentially expressed genes (false discovery rate (FDR) value < 0.01) were selected for further analysis. Heatmaps were generated using Cluster 3.0 and Treeview 1.1.6 based on reads per kb of transcript per million mapped reads (RPKM) values [14, 15].

### Co-immunoprecipitation (co-IP) assay

Human full-length HO-1 cDNA was amplified from a whole human cDNA library and was cloned into PCAGGS-HA with specific primers (Table S3). The recombinant pCMV-C-Flag-CYP1A1 plasmid was established and stored in our laboratory. The pCMV-C-Flag-CYP1A1-edited plasmid was constructed using CYP1A1-edited-F/R primers (Table S3). Two edited versions of CYP1A1 plasmids transfected cells were harvested 24 h post-transfection and lysed with cell lysis buffer (Beyotime, Shanghai, China) supplemented with 1 mM of the protease inhibitor phenylmethylsulfonyl fluoride (PMSF). Following centrifugation, the supernatant was incubated with 1 μg of anti-Flag antibody along with protein G magnetic beads (Invitrogen, Carlsbad, CA, USA) for a duration of 20 min at room temperature. After six washes, the immunoprecipitates were eluted by boiling the beads in sodium dodecyl sulfate (SDS) protein loading buffer for 10 min and subsequently subjected to western blot analysis using corresponding antibodies. IgG was utilized as a negative control.

### ROS measurement

ROS levels were determined by dichlorodihydrofluorescein diacetate (H_2_DCFDA) fluorescence-activated cell sorting (FACS). Oxidation of H_2_DCFDA by intracellular ROS leads to the formation of 2′,7′-dichlorodihydrofluorescein (DCF), which is highly fluorescent and was assessed by FACS analysis. Cells incubated with H_2_O_2_ (500 μM) for 5 min were utilized as a positive control. Experiments were performed in triplicates, and data are presented as mean ± standard deviation (SD).

### Immunofluorescence

Cells grown on coverslips were fixed with 4% formaldehyde and permeabilized by 0.1% Triton X-100. After blocking with 5% bovine serum albumin in phosphate-buffered saline for 1 h, cells were incubated with rabbit anti-HO-1 (Abcam, ab189491, 1:500) in phosphate-buffered saline containing 1% bovine serum albumin for 2 h at room temperature. Cells were incubated with Alexa Fluor 488-conjugated goat anti-rabbit IgG antibody (Servicebio, GB25301, 1:500). Cells were counterstained with 4,6-diamidino-2-phenylindole and examined by a fluorescence microscope (RVL-100, Echo Revolve, USA).

### Subcellular fractionation

Nuclear and cytoplasm fractions were prepared utilizing the Minute™ Cytoplasmic and Nuclear Extraction Kit (Invent Biotech, Minnesota, USA) according to the manufacturer’s instructions.

### LC–MS/MS analysis

The protein samples were prepared using the filter-aided sample preparation (FASP) method and digested with trypsin. Subsequently, the peptides were desalted according to the manufacturer’s instructions. For liquid chromatography tandem mass spectrometry (LC–MS/MS) analysis, 1.5 μg of desalted peptides were loaded onto a Vanquish nanoscale liquid chromatography system coupled with an Orbitrap Exploris 480 mass spectrometer (Thermo scientific, Germany). The elution of peptides was achieved by employing mobile phase A containing 0.1% formic acid and mobile phase B consisting of 80% acetonitrile with 0.1% formic acid. The flow rate was set at 300 nL/min through a 200 mm × 75 μm column packed with 3 μm C18-AQ particles. Our methodology was anchored by the stringent selection of proteins, necessitating the identification of at least two unique peptides per protein and validation across a minimum of two out of three tandem mass spectrometry (MS/MS) quantitative runs. To discern the differential proteins that could underpin phenotypic variations, we adopted a fold change threshold of greater than ± 1.5, coupled with a statistical significance cutoff of *p*-value < 0.05.

### Western blot

The proteins in cells and tissues were extracted with radioimmunoprecipitation assay (RIPA) lysis buffer (Thermo Fisher, Germany). Serum proteins were extracted with a Serum Protein Extraction Kit (BestBio, China), and the concentrations were measured by a bicinchoninic acid (BCA) protein assay kit (Thermo Fisher, Germany). The following antibodies (1:1000) were used: anti-GAPDH (Bioss, bs-10900R), anti-ADAR1 (Bioss, bs-2168R), anti-ADAR2 (Sigma-Aldrich, SAB1405426), anti-β-actin (Cell Signaling Technology, 4967), anti-AKT (Cell Signaling Technology, 9272), anti-Phospho-Akt (Cell Signaling Technology, 9271), anti-Flag (Abcam, ab205606), anti-HA (Abcam, ab236632), anti-CYP1A1 (Abcam, ab235185), and anti-HO-1 (Abcam, ab189491). The immunocomplexes were detected with ECL Western Blotting Substrate (Thermo Fisher, Germany), visualized with BIO-RAD (BIO-RAD, Hercules, CA, USA).

### Statistical analysis

The statistical analyses were conducted using SPSS 20.0 (SPSS, Chicago, IL, USA), GraphPad Prism 8.0 (GraphPad Software Inc, CA, USA), and R software (version 3.6.1). The mean ± SD of at least three independent experiments is reported for all in vitro experiments. Differences between groups were analyzed using Student’s *t*-test, Wilcoxon signed rank test, the Mann–Whitney *U* test, or one-way analysis of variance (ANOVA), correspondingly. Kaplan–Meier survival curves were compared using the log-rank test with GraphPad Prism software. Statistical significance was defined as *p*-value < 0.05.

## Results

### CYP1A1 overediting is associated with NSCLC carcinogenesis

**Fig. 1:**
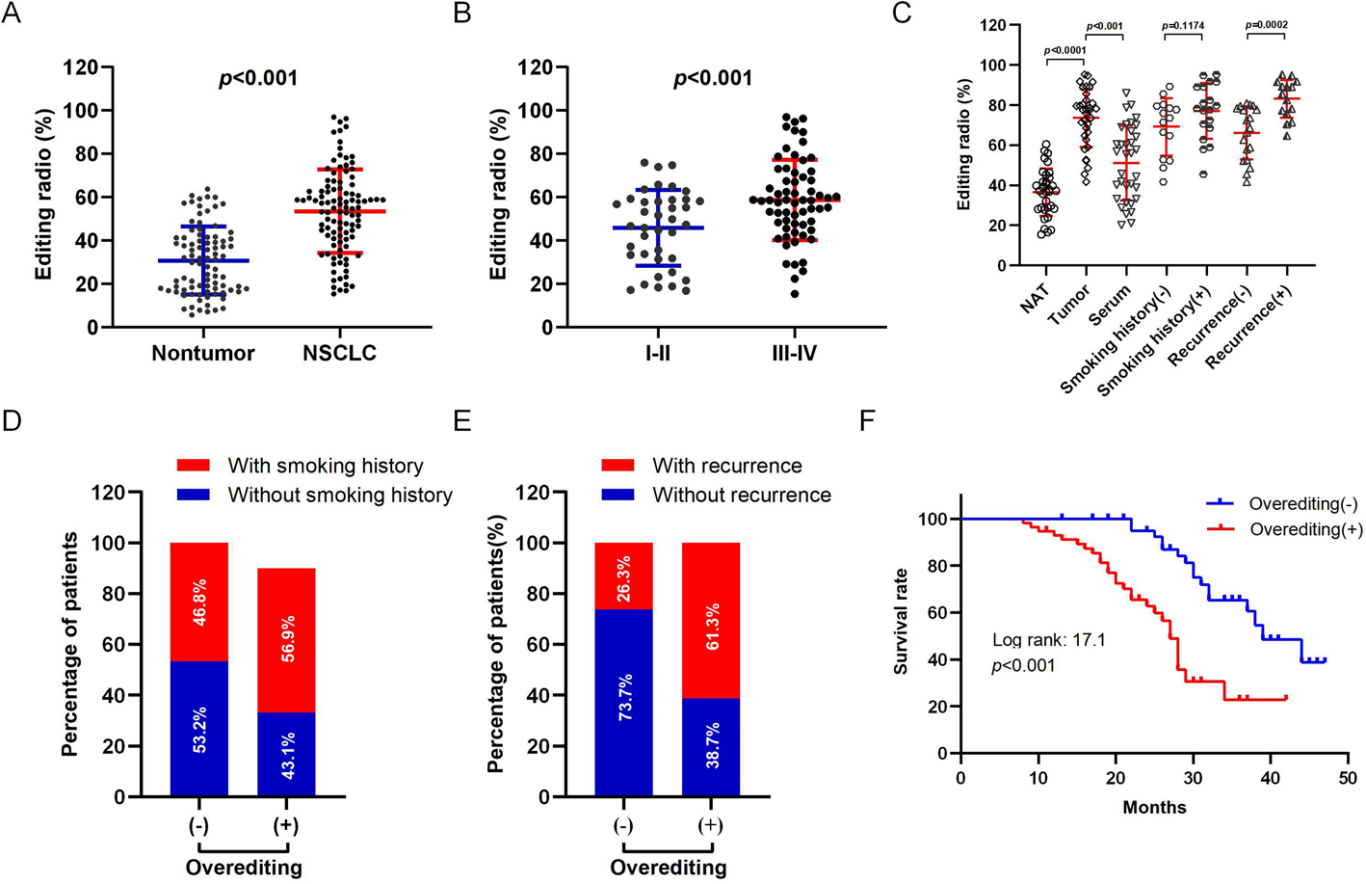
CYP1A1 overediting is strongly associated with NSCLC pathogenesis. **A** CYP1A1 editing of serum samples from patients with NSCLC and non-NSCLC cohorts. **B** The CYP1A1 editing level in a cohort of 103 NSCLC patients stratified by stage. **C** Dot plots showing CYP1A1 editing in serum samples, primary NSCLC, and normal adjacent tissues (NATs) from 103 patients. The *p* values shown were calculated by Mann–Whitney *U* test. The matched NSCLC specimens were subdivided into four categories according to the presence or absence of smoking history or tumor recurrence. Association between smoking history (**D**) or recurrence incidence (**E**) with CYP1A1 overediting (*χ*^2^ test). (**F**) Kaplan–Meier plots for the disease-free survival rate of patients with NSCLC in the groups with (+) and without (−) CYP1A1 overediting. The *p* value shown was calculated by log rank test

Single-nucleotide variations (SNVs) of CYP1A1 at position 4885 have been detected in various lung cancer cell lines using Sanger sequencing (Fig. S1A). Our findings indicated that CYP1A1 exhibited higher A-to-I editing levels in 95D highly metastatic lung cancer cell compared with other cell lines (Fig. S1B), suggesting a potentially pivotal role of this A-to-I RNA edited site of CYP1A1 in cancer progression. To investigate the impact of overedited CYP1A1 during neoplastic transformation, we examined the editing levels of CYP1A1 in human serum samples from patients with NSCLC and non-NSCLC cohorts. The results revealed that CYP1A1 editing levels were dramatically upregulated in NSCLC groups compared with that in non-NSCLC controls (Fig. 1A). Furthermore, we observed a positive association between higher CYP1A1 editing levels and more advanced tumor stage (Fig. 1B; Table S1). Additionally, we detected the CYP1A1 editing levels in 34 matched primary NSCLC and normal adjacent tissues (NATs) from individuals within our cohort, revealing that approximately 61.8% (21/34 patients) of the primary NSCLC specimens exhibited CYP1A1 overediting as defined by an increase of not less than 10% editing in tumors compared with in adjacent nontumor specimens (Fig. 1C). Furthermore, we observed a significant increase in CYP1A1 editing levels of NSCLC tissues compared with that in serum samples (*p* < 0.001) (Fig. 1C). Notably, the extent of CYP1A1 editing level gradually increased during NSCLC pathogenesis from NATs to clinically verified NSCLC (Fig. 1C). Patients with NSCLC tumor recurrence had a higher frequency of CYP1A1 editing compared with those without tumor recurrence (Fig. 1C). Clinicopathological analysis determined that CYP1A1 overediting in tumors was significantly correlated with tumor recurrence (*p* = 0.0002) and worse prognoses (*p* < 0.001) while uncorrelated with smoking history (*p* = 0.1174) (Fig. 1D–F; Table. S2). Collectively, the proportion of edited CYP1A1 increased markedly and showed a correlation with NSCLC progression.

### ADAR1 is responsible for CYP1A1 A-to-I RNA editing

**Fig. 2:**
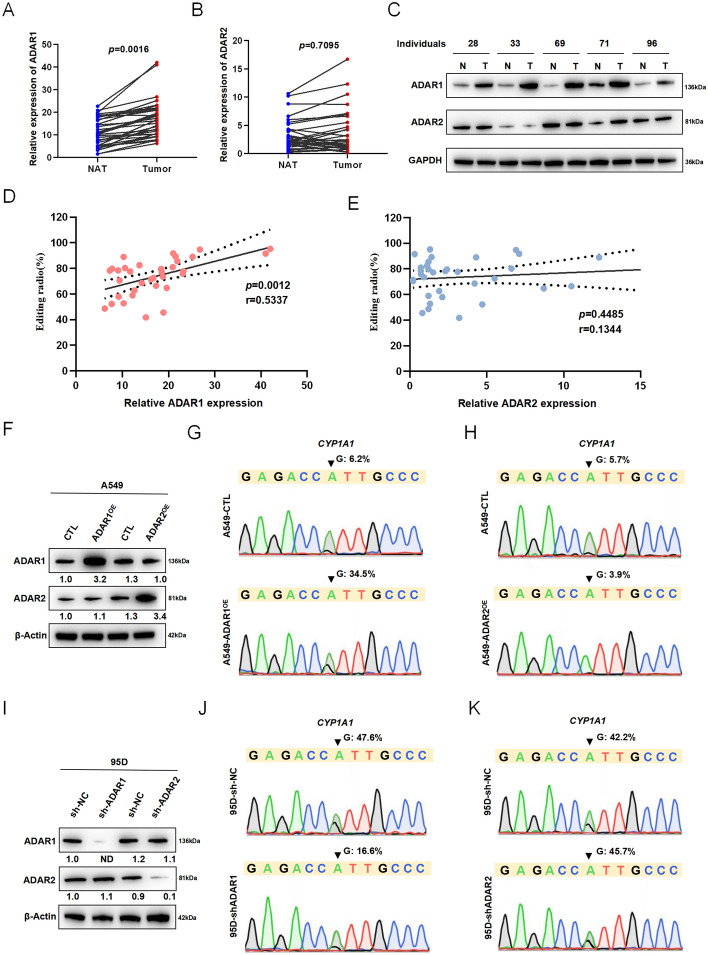
ADAR1 directs CYP1A1 A-to-I RNA editing. Relative expression of ADAR1 (**A**) and ADAR2 (**B**) between NSCLC tissues (Tumor) and matched normal adjacent tissues (NAT), and normalized expression levels of target genes were calculated relative to GAPDH using the ΔΔCT method (Wilcoxon signed rank test). **C** Western blot showing expression of ADAR1 and ADAR2 proteins in NSCLC tissues (T) and matched normal adjacent tissues (N) specimens from individuals 28, 33, 69, 71, and 96. GAPDH was the loading control. Correlations between CYP1A1 editing levels and the relative normalized mRNA levels of ADAR1 (**D**) and ADAR2 (**E**) of 34 paired specimens. Solid lines represent the linear regression (*r*), and dashed lines represent the 95% confidence interval (Spearman correlation coefficient test). **F** Western blot showing the expression of ADAR1 and ADAR2 proteins in A549 cells transiently transfected with the indicated expression constructs. CTL, control construct. β-Actin was the loading control. **G**, **H**, **J**, **K** Sequence chromatograms of the CYP1A1 transcript in the indicated cell lines; the arrowheads indicate the edited positions. (**I**) Western blot of ADAR1 and ADAR2 in 95D cells transiently transfected with expression plasmids as indicated. sh-NC, negative control; sh-ADAR1, shRNA specific to ADAR1; sh-ADAR2, shRNA specific to ADAR2. Values above each band represent band intensity and were calculated using ImageJ software. Band intensity in the first lane was normalized as 1.0. ND, not detected

A-to-I RNA editing is catalyzed by ADAR enzymes, which comprise three structurally conserved members: ADAR1, ADAR2, and ADAR3. Since ADAR3 had no documented catalytic activity [16], our focus was directed toward ADAR1 and ADAR2 to study their role in CYP1A1 A-to-I RNA editing. The relative expression of both ADAR1 and ADAR2 was examined in clinical specimens from 34 patients with NSCLC. The results revealed significantly higher expression of ADAR1 in cancerous tissues compared with NATs (*p* = 0.0016) (Fig. 2A), while no significant difference was observed in the relative expression of ADAR2 between tumor and matched nontumor tissues (*p* = 0.7095) (Fig. 2B). Further analysis demonstrated a significantly enhanced expression of lung cancer tissue-specific ADAR1 compared with normal adjacent tissues in five paired specimens, whereas no obvious difference was observed for ADAR2 in these matched specimens (Fig. 2C). Additionally, a positive correlation was observed between the relative normalized quantification value and the editing level of CYP1A1 for ADAR1 but not for ADAR2 (Fig. 2D, E).

To explore whether there is a major role played by ADAR1 in CYP1A1 A-to-I editing, overexpression experiments were conducted separately for ADAR1 and ADAR2 in A549 cells, followed by assessment of the CYP1A1 editing level through Sanger sequencing analysis (Fig. 2F). As depicted in Fig. 2G, H, overexpression of ADAR1 resulted in an approximate sixfold increase in CYP1A1 editing, whereas overexpression of ADAR2 showed no significant effect on its editing level. Meanwhile, silencing experiments targeting ADAR1 were performed on NSCLC cell line 95D, which led to a marked decrease in CYP1A1 editing levels; conversely, knockdown of ADAR2 did not exhibit any significant change in CYP1A1 editing levels compared with the negative control cells (Fig. 2I–K). The studies of expression of ADAR family members and functional identification support the notion that ADAR1 plays a more critical role than ADAR2 in CYP1A1 A-to-I RNA editing.

### CYP1A1 A-to-I RNA editing promotes the proliferation, invasion, and migration of NSCLC cells

**Fig. 3:**
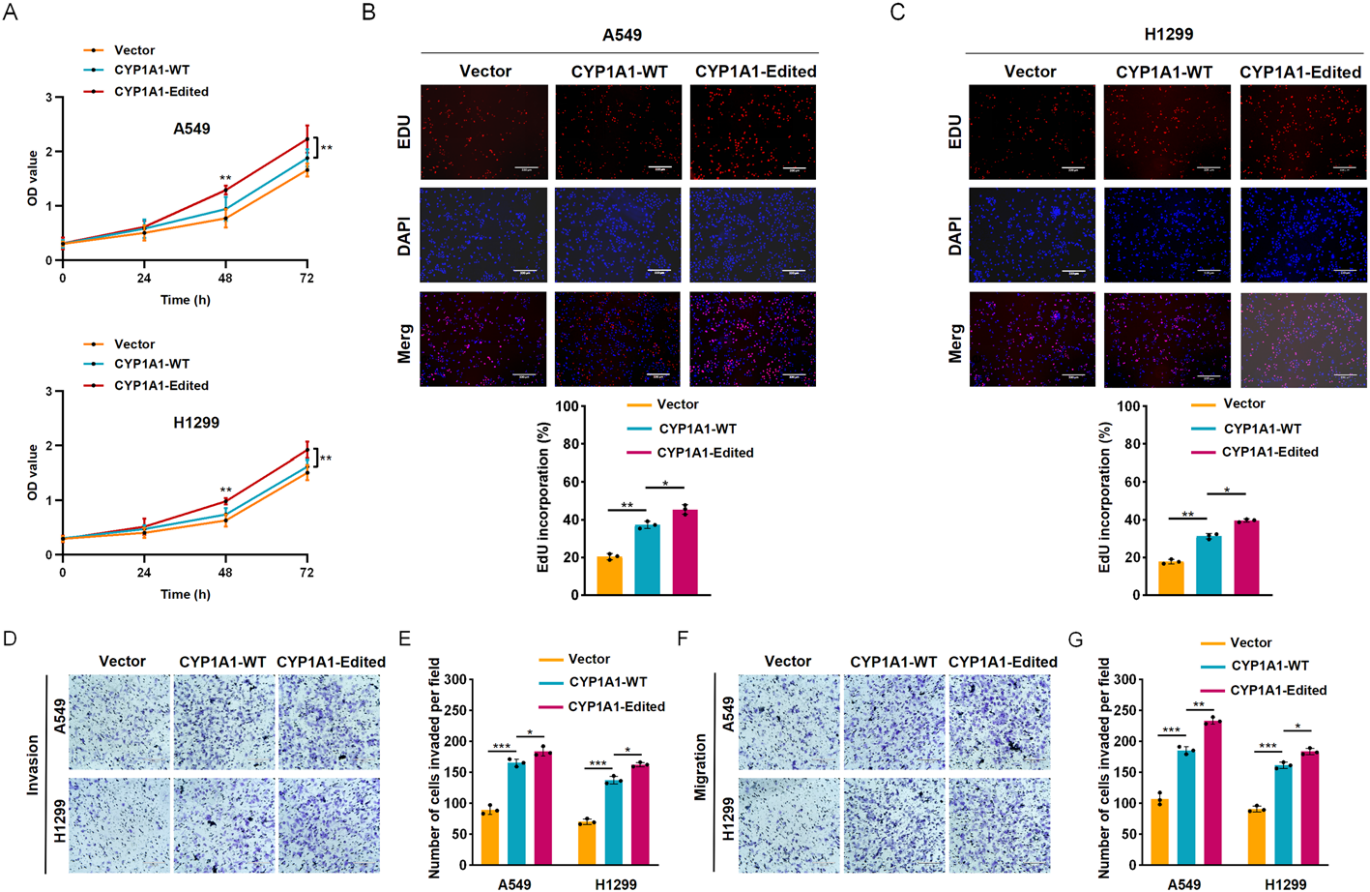
CYP1A1 A-to-I RNA editing confers enhanced tumorigenicity. **A** The growth rates of the indicated stable cell lines was measured by CCK-8. OD, optical density. EdU assays revealed that CYP1A1 A-to-I RNA editing enhanced the proliferation ability of indicated A549 (**B**) and H1299 (**C**) cells. Scale bars: 330 μm. The invasion (**D**, **E**) and migration abilities (**F**, **G**) of indicated stable cell lines were enhanced by CYP1A1 A-to-I RNA editing through Transwell assays. Scale bars: 220 μm. Scale bars: 220 μm. Standard deviation (SD) of three independent experiments. One-way ANOVA with Tukey’s test as post hoc test was used to assess the difference; *p < 0.05; **p < 0.01; ***p < 0.001

To ascertain the potential association between functional phenotypes in cancer development and CYP1A1 A-to-I RNA editing, we introduced Flag-tagged wild-type CYP1A1 and edited CYP1A1 (CYP1A1_I462V) expression constructs into A549 and H1299 cell lines, respectively. The protein expression level of the exogenously introduced edited CYP1A1 was similar to that of the wild-type CYP1A1, as confirmed by western blot (Fig. S2A). The editing frequency of CYP1A1 was determined using Sanger sequencing, revealing a marked increase in editing frequency when edited CYP1A1 was overexpressed (Fig. S2B). Subsequently, we found that edited CYP1A1 overexpression significantly enhanced the proliferative capacity of both A549 and H1299 cells compared with the WT CYP1A1 and control group through CCK-8 assays, and EdU assays (Fig. 3A–C). Additionally, Transwell assays indicated that the invasion and migration abilities of A549 and H1299 cells were remarkably strengthened by the overexpression of edited CYP1A1 (Fig. 3D–G).

To further confirm the causal relationship between gain-of-function phenotypes and edited CYP1A1-dependent manner, we utilized the CRISPR/Cas9 system to introduce a point mutation occurs at residue 462 of CYP1A1 in A549, resulting in an isoleucine to valine amino acid substitution, thereby generating the A549^I462V^ mutant cell line. As expected, 100% editing was detected in the gDNA samples of CYP1A1 in A549^I462V^ (Fig. S3A). Subsequently, we assessed the cell proliferative capacity of A549 and A549^I462V^ cells using EdU assays. A quantitative evaluation revealed that A549^I462V^ cells showed a significantly increased proliferation rate compared with A549 cells (Fig. S3B). Moreover, A549^I462V^ cells could boost the invasive and migratory phenotype through Transwell assays and wound healing assays, in comparison with control cell A549 (Fig. S3C, D). Taken together, our results suggest that CYP1A1 A-to-I RNA editing may exert an oncogenic role in NSCLC.

### CYP1A1 A-to-I RNA editing promotes tumorigenic phenotype in vivo

**Fig. 4:**
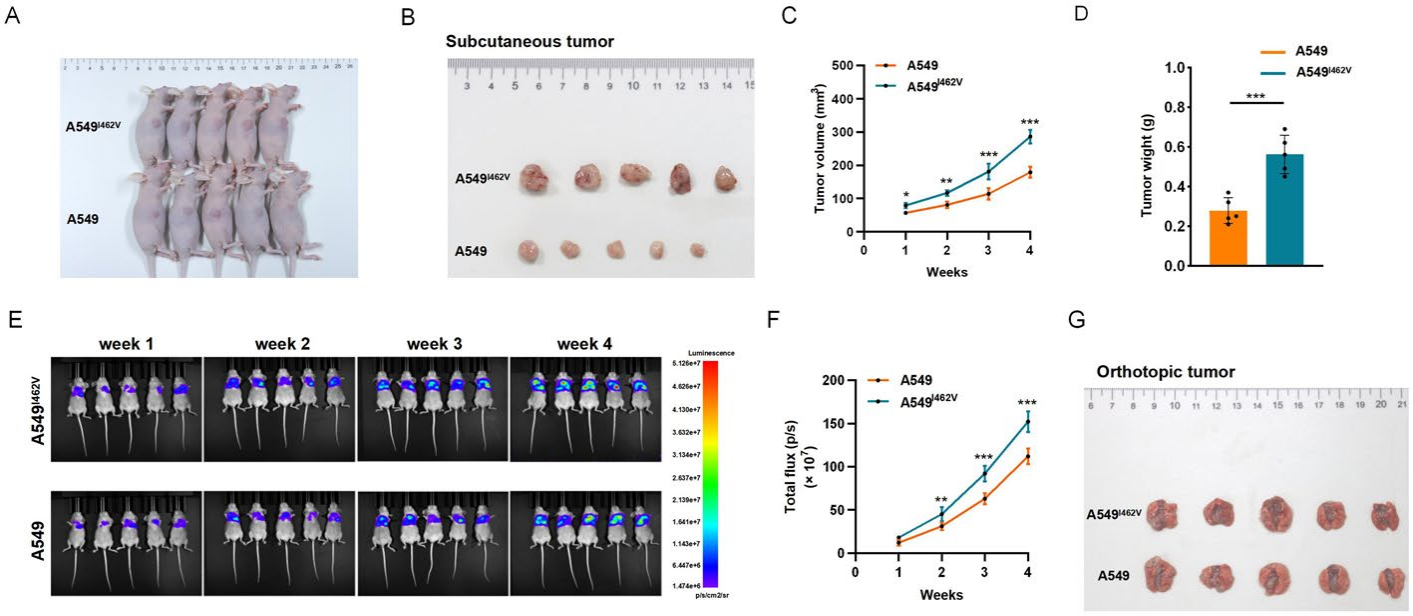
CYP1A1 overediting promotes the growth of NSCLC cells in vivo. **A**, **B** Images of xenograft tumors after injection of A549 cells or A549^I462V^ cells (*n* = 5 per group). **C** The volumes of subcutaneous tumors were recorded once a week for four consecutive weeks. **D** The subcutaneous tumor weights were weighed at the endpoint time of the experiment. **E** The orthotopic tumor growth of lung was monitored weekly by in vivo bioluminescence imaging (*n* = 5 per group). **F** The bioluminescence signals of orthotopic tumors were measured by mean photon counts. **G** Gross appearance of orthotopic tumor after lung parenchyma at the left lateral dorsal axillary line injection with A549 cells or A549^I462V^ cells (*n* = 5 per group)

To validate the oncogenic role of CYP1A1 A-to-I RNA editing in vivo, both subcutaneous xenograft and orthotopic xenograft models were established. For the subcutaneous xenograft model, nude mice were randomly divided into two groups (*n* = 5 per group). The results showed that tumor growth was remarkably enhanced in the mice injected with A549^I462V^ cells compared with that in the A549 group after 4 weeks (Fig. 4A, B). Tumors induced by A549I462V cells exhibited significantly faster growth than those induced by A549 cells (Fig. 4C, D). The orthotopic xenograft studies in mice demonstrated that nude mice injected with A549I462V cells exhibited stronger bioluminescence signals in comparison with A549 group at each time point (Fig. 4E, F). Furthermore, 4 weeks after intrapulmonary inoculation, mice injected with A549^I462V^ cells formed more lung nodules (Fig. 4G). Subsequently, IHC analysis revealed that injection with A549^I462V^ cells confers enhanced tumorigenicity in mouse orthotopic tumor tissues compared with A549 cells (Fig. S4A–C). Collectively, our data suggested that CYP1A1 overediting facilitates in vivo tumorigenic ability in NSCLC.

### The effect of CYP1A1 overediting on tumor progression is partially dependent upon the PI3K/AKT signaling pathway

**Fig. 5:**
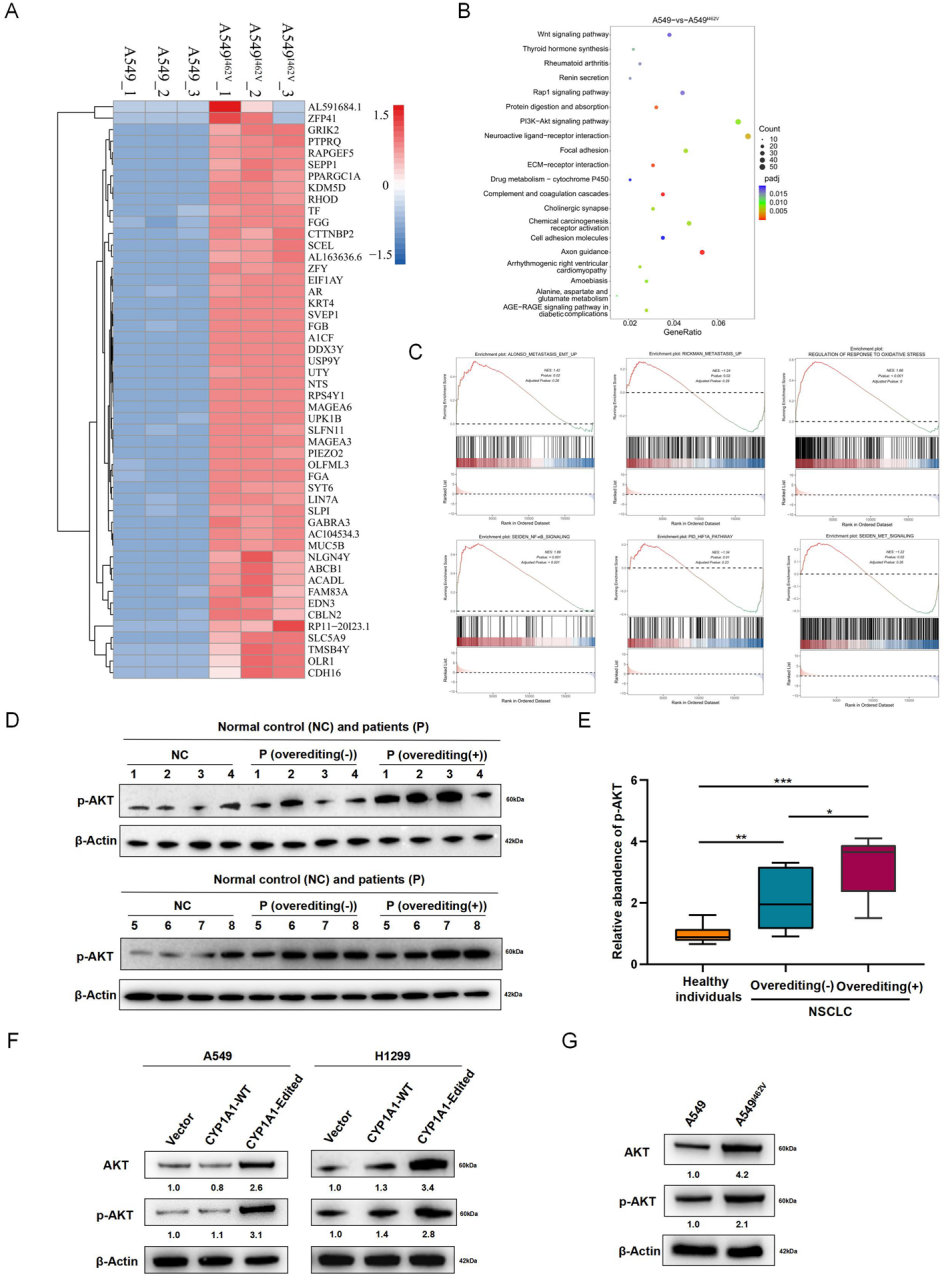
CYP1A1 overediting activated the PI3K/AKT signaling pathways in NSCLC cells. **A** Heatmap illustrating the signature of the top 50 genes upregulated by A549^I462V^ as determined by transcriptome profile analysis. FDR *p* < 0.01. **B** KEGG pathway enrichment analyses of DEGs between A549 and A549^I462V^ cells. **C** GSEA of the A549^I462V^ targeted upregulated gene signature. **D** Western blot of total Akt in the serum samples from normal control subjects and patients with lung cancer with CYP1A1 overediting or not. β-Actin was used as the internal control. The corresponding quantification data are shown in **E**. **F** Western blot showing expression level of total Akt and phospho-Akt in both A549 and H1299 cells after transfection with different types of CYP1A1. **G** Western blot analysis of total Akt and phospho-Akt in A549 and A549^I462V^ cells. Values above each band represent band intensity and were calculated using ImageJ software. Band intensity in the first lane was normalized as 1.0. One-way ANOVA with Tukey’s test as post hoc test was used to assess the difference; *p < 0.05; **p < 0.01; ***p < 0.001

To investigate the potential role of CYP1A1 A-to-I RNA editing in tumorigenic properties in NSCLC, RNA-seq was performed to compare A549 and A549^I462V^ cells to further elucidate the involvement of CYP1A1 overediting in tumor progression (Fig. 5A). Prior to analyzing the RNA-seq profiles, reproducibility was evaluated in three replicate experiments using linear correlation analysis. The high correlation coefficients (*r* = 0.993, 0.996, and 0.998) between the replicates indicated the reliability of the RNA-seq data under the experimental conditions. Kyoto Encyclopedia of Genes and Genomes (KEGG) pathway analysis indicated that the differentially expressed genes (DEGs) between A549 and A549^I462V^ cells were significantly associated with the PI3K-Akt signaling pathway (Fig. 5B). Additionally, gene set enrichment analysis (GSEA) revealed obvious enrichment in metastasis-related pathways, which were positively associated with the recurrence and prognosis of malignant tumors (Fig. 5C). Although hyperactivation of the PI3K-Akt signaling pathway is one of the most ordinary events in human cancers [17], there is limited research on its relationship with A-to-I RNA editing in NSCLC. The levels of p-Akt in human serum were examined using a clinical sample set, which included serum samples from eight normal control subjects, eight patients with NSCLC without CYP1A1 overediting, and eight patients with NSCLC with CYP1A1 overediting for confirmation. As anticipated, there was a significant difference between CYP1A1 overediting group and the group without CYP1A1 overediting or normal control (CYP1A1 overediting versus CYP1A1 without overediting, *p* = 0.038; CYP1A1 overediting versus normal control, *p* < 0.001) (Fig. 5D, E). Additionally, the levels of total Akt and phospho-Akt in both A549 and H1299 cells transfected with the control vector, WT CYP1A1, and edited CYP1A1 plasmids were further validated. The results indicated that a high level of total Akt and phospho-Akt expression were observed when edited CYP1A1 was introduced, compared with WT CYP1A1 or the control vector, as confirmed by western blot (Fig. 5F). Furthermore, there was a substantial increase in total Akt and phospho-Akt expression level of A549^I462V^ in comparison with A549 control cell (Fig. 5G), suggesting that CYP1A1 overediting enhanced the tumor progression through PI3K-Akt signaling pathway activation.

### CYP1A1 overediting inhibits oxidative stress via activated PI3K/Akt

**Fig. 6:**
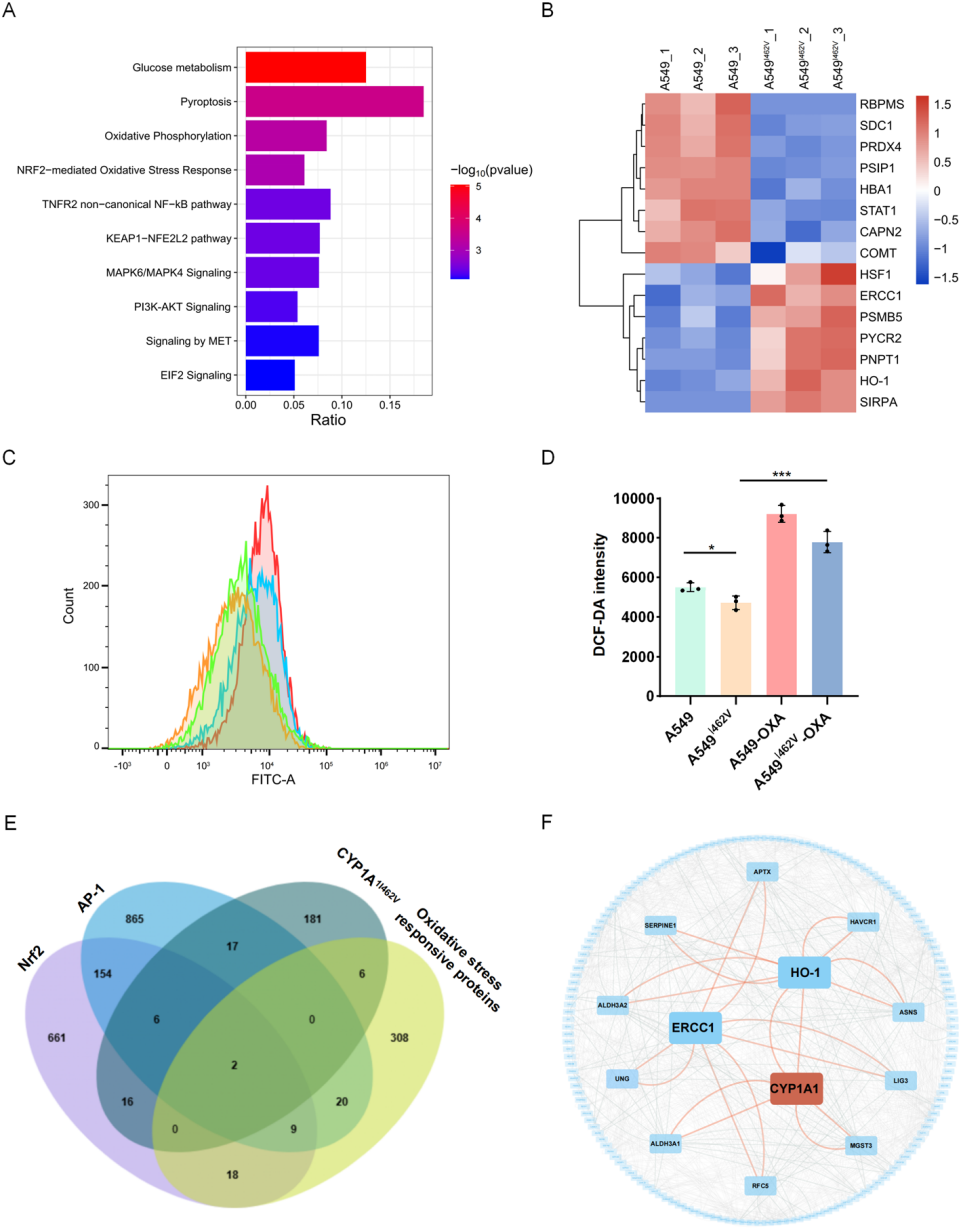
CYP1A1_I462V showed increased tolerance to oxidative stress in NSCLC. **A** KEGG pathways of differentially expressed proteins (DEPs) in A549^I462V^. **B** Heatmap illustrating the top 15 DEPs in A549^I462V^, as determined by proteome profile analysis. **C** Cells stained with 10 μM DCF-DA and analyzed by flow cytometry. Green panel: A549; orange panel: A549^I462V^; red panel treated with 1 μM OXA; blue panel: A549^I462V^ treated with 1 μM OXA. **D** ROS generation was analyzed by Fluorence microplate reader. All results expressed as mean ± SEM of three independent experiments. *p < 0.05; ***p < 0.001. **E** Venn diagram comparing A549^I462V^ − upregulated proteins, oxidative stress responsive proteins, and the target genes of the oxidative stress-related transcription factors Nrf2 and AP-1. **F** Protein–protein interaction (PPI) network of CYP1A1 and the upregulated protein signature as indicated

To clarify the downstream regulatory mechanism of CYP1A1_I462V, we performed proteomic analysis by LC–MS/MS in both A549 and A549^I462V^ cells. KEGG pathway analysis showed a positive correlation between CYP1A1_I462V and the regulation of reactive oxygen species (Fig. 6A). Remarkably, oxidant responsive-related protein HSF1, PYCR1, and HO-1 were significantly upregulated in A549^I462V^ compared with A549 (Fig. 6B). Our results discovered that the mean ROS activity in A549^I462V^ cells was obviously lower compared with that in A549, which revealing that CYP1A1 overediting reduced the accumulation of ROS in A549 (Fig. 6C, D). Moreover, we further explored whether CYP1A1 overediting protected against oxidative injury by activating PI3K/Akt signaling pathway to suppress the intracellular ROS generation. After exposure to AKT inhibitor OXA, a significant increase in ROS production was observed in A549I462V cells (Fig. 6C, D). To further investigate the role of CYP1A1_I462V in oxidative stress response, two upregulated candidates were identified among the sets of A549^I462V^—upregulated proteins, oxidative stress responsive proteins, and target genes of the oxidative stress-related transcription factors Nrf2 and AP-1, including heme oxygenase-1 (HO-1) and excision repair cross-complementation group 1 (ERCC1) (Fig. 6E). The accumulating evidence suggested that Nrf2 induces HO-1 expression via PI3K/Akt-dependent activation in different cancer cells [18, 19]. Interestingly, protein–protein interaction (PPI) network analysis of the upregulated genes in this intersection signature revealed intimate protein interaction between CYP1A1 and HO-1 (Fig. 6F). Thus, we were particularly interested in HO-1 owing to its function of oxidative stress response in the presence of CYP1A1_I462V.

### CYP1A1 overediting enhances the interaction with HO-1 and mediates nuclear localization of HO-1 to resist oxidative stress in NSCLC cells

**Fig. 7:**
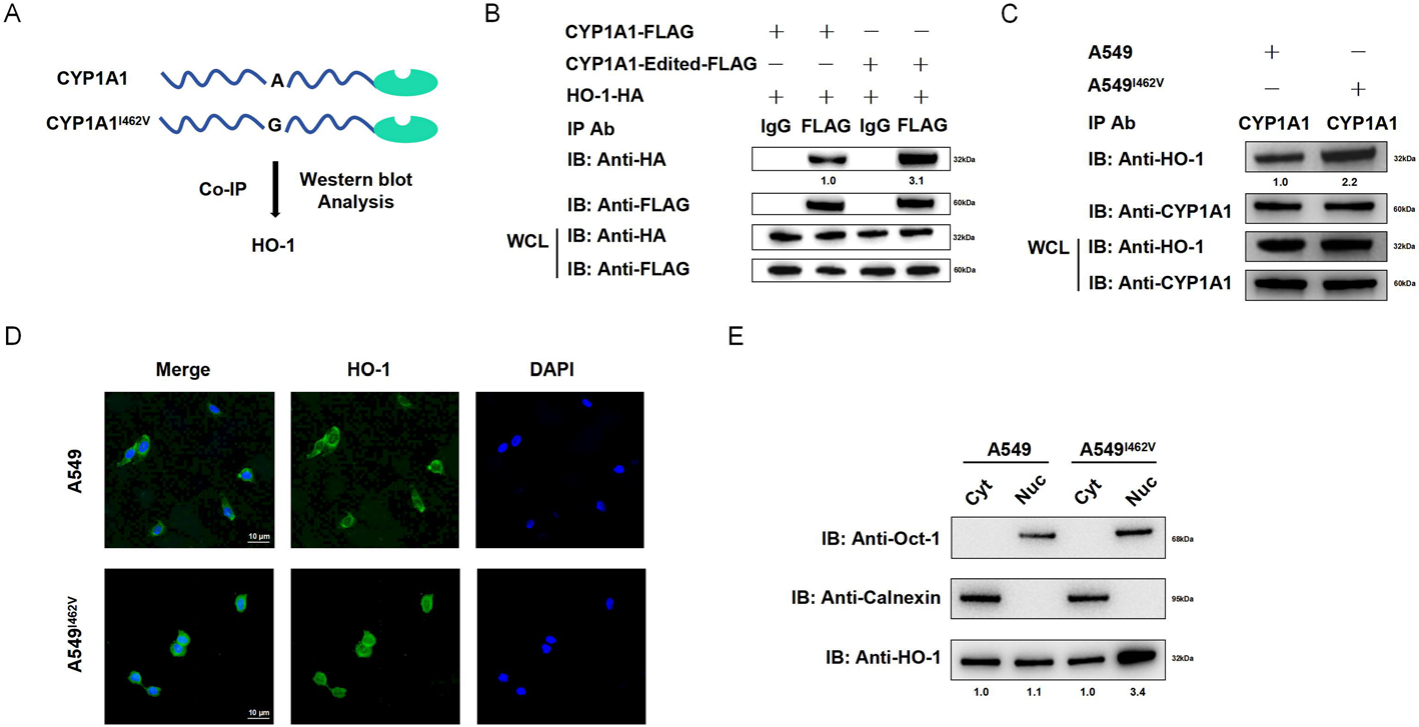
CYP1A1_I462V enhanced the interaction with HO-1 and contributed to HO-1 nuclear localization. **A** Western blot assay was applied in 293T cells to identify the proteins that interacted with CYP1A1. **B** 293T cells were cotransfected with CYP1A1-Flag and HA-HO-1 or CYP1A1-edited-Flag and HA-HO-1. **C** Co-IP assay was applied in A549 or A549^I462V^ cells using CYP1A1 antibody to detect endogenous levels of HO-1 protein. Immunoblot analysis was performed with anti-HA and anti-Flag antibodies to detect the precipitated protein and the expression of transfected protein (WCL). **D** The expression of HO-1 in A549 and A549^I462V^ cells was examined by immunofluorescence. Bar, 10 μm. **E** The levels of HO-1 in nuclear and cytoplasm fractions were examined using western blot analysis in A549 and A549^I462V^ cells. Oct-1 and calnexin served as the markers of nuclear and microsomal fractions, respectively. Nuc: nuclear; Cyt: cytoplasm. Band intensity in the first lane was normalized as 1.0

To explore the molecular mechanism underlying the progression of non-small-cell lung cancer (NSCLC) induced by CYP1A1 overediting, a Flag-tagged CYP1A1-edited probe and a control probe were used to identify the interaction between HO-1 and CYP1A1 or CYP1A1_I462V in 293T cells, respectively, through co-IP assay (Fig. 7A). Given that previous study has reported that HO-1 and CYP1A1 could form a stable complex [20], we hypothesized that the interaction between HO-1 and CYP1A1 would be enhanced in the presence of CYP1A1 overediting. To validate our hypothesis, HA-HO-1 and Flag-CYP1A1 or Flag-CYP1A1-edited constructs were respectively cotransfected into 293T cells. Immunoprecipitation using an anti-Flag antibody, followed by immunoblotting with an anti-HA antibody, revealed strong interaction between CYP1A1-edited and HO-1 in 293T cells (Fig. 7B). Moreover, an endogenous Co-IP assay using an anti-CYP1A1 antibody was performed to further elucidate the relationship between CYP1A1 overediting and its binding affinity to HO-1. We also observed that the expression level of HO-1 was significantly increased in A549^I462V^ cells when using endogenous CYP1A1 protein as bait (Fig. 7C), which indicated that CYP1A1 overediting enhanced its interaction with HO-1. Nuclear translocation of HO-1 is particularly sensitive to cellular stress, such as oxidative stress and hypoxia [21, 22]. Our result demonstrated that HO-1 nuclear localization was prominent in A549^I462V^ cells (Fig. 7D). Furthermore, we performed the subcellular fractionation of A549 and A549^I462V^ cells, which express high levels of endogenous HO-1. As shown in Fig. 7E, western blot analysis revealed that the relative expression of HO-1 exhibited greater abundance in the nuclear compartment compared with the cytoplasm in A549^I462V^ (Fig. 7E).

## Discussion

The edited inosine in A-to-I editing is recognized as guanosine during posttranscriptional alteration of RNA sequences, resulting in amino acid substitution. This confers the ability to adapt to the complex environment both inside and outside the body on the RNA-editing process [23, 24]. Abnormalities in RNA editing have been observed in numerous coding and noncoding genes associated with various human malignant tumors. These abnormalities contribute to the acquisition of tumorigenic characteristics, cancer cell invasion, and metastasis [25, 26]. In this study, we identified CYP1A1 overediting as an important modifying factor that determined susceptibility to NSCLC. Furthermore, we detected the accumulation of an I462V substitution in tumor specimens.

CYP1A1 is considered as an extrahepatic isoform, predominantly expressed in the intestine, lung, brain, placenta, and kidney; however, it can also be induced in the liver [27]. Herein, we observed a higher frequency of CYP1A1 editing in lung tissues compared with serum samples from patients. This finding confirmed the physiological significance of CYP1A1 editing specifically within lung tissue, which aligned with previous reports [28]. Sanger sequencing analysis of 34 paired NSCLC specimens revealed that the level of site-specific CYP1A1_I462V editing was significantly elevated in NSCLC tissues compared with their matched NATs. These results suggest an increased level of CYP1A1 editing during the multistep progression of NSCLC. Furthermore, overediting of CYP1A1 in tumors was found to be predictive of poor prognosis and associated with tumor recurrence independent of smoking history. Interestingly, conflicting epidemiological studies have reported varying correlations between genetic polymorphisms in CYP1A1 and smoking status among NSCLC patients [29, 30]. Additionally, alterations in expression levels for ADAR (the A-to-I RNA-editing regulator) have been observed across different types of cancer [31, 32]. Our findings demonstrated that RNA editing of CYP1A1 was specifically regulated by ADAR1, but not by ADAR2. Moreover, we observed a higher level of CYP1A1 RNA editing in the highly metastatic lung cancer cell line 95D compared with other cell lines, which suggested that there could be a positive correlation between CYP1A1 overediting and tumor progression. A series of assays conducted in both cell culture and xenograft studies provided direct evidence that RNA editing (I462V) conferred an enhanced tumorigenic ability, as evidenced by increased proliferation stimulation, greater invasive ability, and higher incidence of tumor formation. Collectively, these data demonstrated that CYP1A1_I462V RNA editing plays an active role in the development of tumorigenic phenotypes.

Cytochrome P450s are heme-containing enzymes that catalyze various phase I metabolism reactions [33]. Among the CYP1 family members, P450 CYP1A1 is known to activate different compounds into reactive forms, thereby enhancing downstream signaling and potentially contributing to carcinogenesis [34, 35]. Previous studies have supported that CYP1A1 polymorphisms played a role in the pathogenesis of lung cancer [36, 37]. However, the oncogenic molecular mechanism underlying RNA editing abnormalities remains poorly understood. Here, KEGG enrichment analysis indicated that RNA editing of I462V in CYP1A1 was involved in the PI3K/Akt signaling pathway. On the basis of previous findings linking CYP1A1 to the PI3K-Akt signaling pathway in breast and prostate cancer [34, 38], we observed that the CYP1A1_I462V mutant increased both total Akt levels and Akt phosphorylation in cells expressing edited CYP1A1 constructs as well as A549^I462V^ cell lines. These results suggested that the I462V mutant of CYP1A1 may act as an upstream activator of the PI3K-Akt signaling pathway in NSCLC.

HO-1, a rate-limiting enzyme responsible for catalyzing the oxidative degradation of cellular heme to release free iron, is involved in cancer and stimulates tumor progression through various mechanisms, including immune suppression, angiogenesis, and metastasis [39]. Furthermore, studies have shown that the activated PI3K/Akt signaling pathway enhances the production of HO-1 by upregulating Nrf2 expression [40]. However, HO-1 does not function independently; it requires physical interaction with cytochrome P450 reductase (POR) for its activity [20]. As both HO-1 and CYP1A1 co-reside in the endoplasmic reticulum membrane [41], we examined that HO-1 and CYP1A1 showed a significantly higher complex stability with each other in the presence of I462V RNA editing of CYP1A1, which may result in effective competition between HO-1 and POR for functional performance. Therefore, it can be inferred that CYP1A1 may have different effects on regulating the activation of HO-1 depending on CYP1A1_I462V RNA editing. Efforts are underway to identify the binding region of the interaction between CYP1A1 and HO-1 to fully understand its role in tumor progression in our future study.

An overload level of reactive oxygen species (ROS) can oxidize DNA, RNA, proteins, and lipids, causing irreversible damage and serious oxidative stress that eventually provoke cell death [42]. Our results further confirmed that HO-1 protects cells by diminishing oxidative stress in NSCLC, thereby promoting cell survival, which was consistent with previous study [43]. Normally, after synthesis, the HO-1 protein is delivered to and anchored at the smooth endoplasmic reticulum (SER) membrane [44]. HO-1 can be trafficked to the mitochondria, nucleus, and rigid domains in the plasma membranes under stress or disease conditions [45]. Our results demonstrated that abundant HO-1 was detected to combine with CYP1A1_I462V and then transferred into the nucleus to resist oxidant stress. However, the mechanism of how HO-1 is delivered into the nucleus in the condition of CYP1A1_I462V RNA editing in NSCLC remains to be elucidated in further study.

## Conclusions

Our research provides valuable insights into the regulation of epigenetic mechanisms in NSCLC initiation and progression mediated by A-to-I RNA editing (graphical abstract). Activated HO-1 contributes to oncogenic signaling, and its high expression level predicts poor prognosis, suggesting that inhibition of HO-1 may be an effective target for cancer therapy. Our work delineates the underlying molecular mechanisms of CYP1A1_I462V RNA editing-regulated activation of HO-1 in NSCLC. Thus, further study is required to investigate the hypothesis that CYP1A1 may act as a new potential therapeutic target for various types of HO-1-activated carcinomas.

## Supplementary Information

Supplementary Material 1: Fig. S1. (A) Sequence analysis of CYP1A1 in edited regions from different lung cancer cell lines, such as A549, H1299, H460, 95C, and 95D. PCR fragments were obtained from cDNA (mRNA) using primers described in “Methods.” Samples were subjected to Sanger sequencing. (B) The CYP1A1 editing level of each cell line. Fig. S2. (A) Western blots of CYP1A1 overexpression in A549 and H1299 cells. Flag tag was used to quantify the CYP1A1 protein expression level, and GAPDH was used as loading control. (Control: control vector, WT: CYP1A1 wild-type, Edited: CYP1A1 edited-type). (B) Changes in CYP1A1 editing level after transfection with different types of CYP1A1 by Sanger sequencing. Three independent biological replicates were carried out in this experiment. One-way ANOVA with Tukey’s test as post hoc test was used to assess the difference; *** < 0.001. Fig. S3 .(A) Sequence chromatograms was detected in gDNA samples of A549 and A549I462V cells. (B) Assessment of the proliferation of A549 and A549I462V cells by EdU assays. (C) Wound healing of A549 and A549I462V cells. Scale bars, 550 μm. (D) The invasion and migration abilities of A549 and A549I462V cells. Scale bars, 210 μm. Standard deviation (SD) of three independent experiments. One-way ANOVA with Tukey’s test as post hoc test was used to assess the difference; **, *p* < 0.01; ***, *p* < 0.001. Fig. S4. (A, B) Images of HE and IHC staining by anti-PD-L1 antibody in orthotopic tumors (*n* = 5 per group). Scale bars: 50 μm. (C) H-score of Ki67 in orthotopic tumors, standard deviation (SD) of three independent experiments. One-way ANOVA with Tukey’s test as post hoc test was used to assess the difference. **, *p* < 0.01.

Supplementary Material 2.

## Data Availability

The datasets generated and/or analyzed during the current study are available from the corresponding author on reasonable request.

## Abbreviations

NSCLC: Non-small-cell lung cancer
CYP1A1: Cytochrome p450 1A1
HO-1: Heme oxygenase-1
ERCC1: Excision repair cross-complementation group 1
A-to-I: Adenosine to inosine
ADAR: Double-stranded RNA-specific adenosine deaminase
UTRs: Untranslated regions
HCC: Hepatocellular carcinoma
ODC: Ornithine decarboxylase
CCND1: Cyclin D1
SNPs: Single-nucleotide polymorphisms
SNVs: Single-nucleotide variations
sgRNA: Single-guide RNA
NATs: Normal adjacent tissues
DEGs: Differentially expressed genes
ROS: Reactive oxygen species
Co-IP: Co-immunoprecipitation
CRISPR/Cas9: Clustered regularly interspaced short palindromic repeats/Cas9
qRT-PCR: Quantitative real-time PCR
GAPDH: Glyceraldehyde 3-phosphate dehydrogenase
PMSF: Phenylmethylsulfonyl fluoride
H_2_DCFDA: Dichlorodihydrofluorescein diacetate
FACS: Fluorescence-activated cell sorting
LC–MS/MS: Liquid chromatography tandem mass spectrometry
RIPA: Radioimmunoprecipitation
BCA: Bicinchoninic acid
ANOVA: Analysis variance
KEGG: Kyoto encyclopedia of genes and genomes
GSEA: Gene set enrichment analysis
