# Correction: A novel mechanism for A-to-I RNA-edited CYP1A1 in promoting cancer progression in NSCLC

**DOI:** 10.1186/s11658-025-00755-1

**Published:** 2025-07-10

**Authors:** Zhipeng Wang, Yan Wu, Ziqi Ding, Xinru Xiao, Yanhua Huang, Zhiguang Liu, Qian Zhang

**Affiliations:** 1 Department of Respiratory and Critical Care Medicine, The Second People’s Hospital of Changzhou, The Third Affiliated Hospital of Nanjing Medical University, Changzhou, 213164 China; 2 Changzhou Medical Center, Nanjing Medical University, Changzhou, 213164 China; 3 State Key Laboratory of Microbial Metabolism, Joint International Research Laboratory of Metabolic and Developmental Sciences, School of Life Sciences and Biotechnology, Shanghai Jiao Tong University, Shanghai, 200240 China

**Correction to: Cellular & Molecular Biology Letters (2025) 30:40** 10.1186/s11658-025-00718-6

In this article [1], there was a minor error in Fig. 3. In Fig. 3F, the control vector of migration in A549 and H1299 were mixed up. For completeness and transparency, the old incorrect and correct versions are displayed in this correction.

Incorrect Fig. 3:

**Fig. 3:**
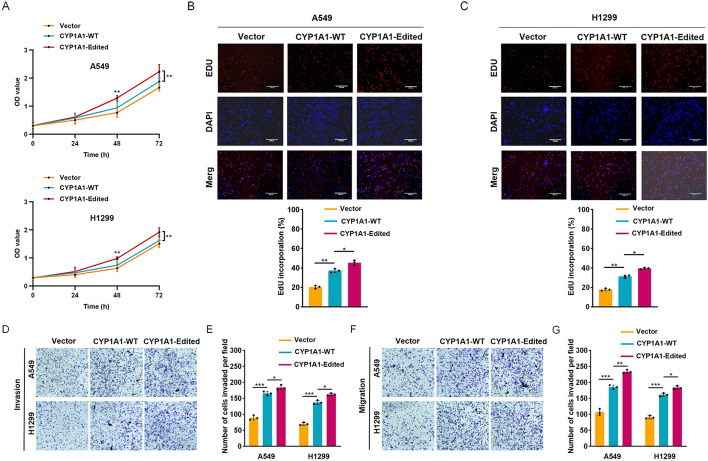
CYP1A1 A-to-I RNA editing confers enhanced tumorigenicity. **A** The growth rates of the indicated stable cell lines was measured by CCK-8. OD, optical density. EdU assays revealed that CYP1A1 A-to-I RNA editing enhanced the proliferation ability of indicated A549 (**B**) and H1299 (**C**) cells. Scale bars: 330 μm. The invasion (**D, E**) and migration abilities (**F, G**) of indicated stable cell lines were enhanced by CYP1A1 A-to-I RNA editing through Transwell assays. Scale bars: 220 μm. Scale bars: 220 μm. Standard deviation (SD) of three independent experiments. One-way ANOVA with Tukey’s test as post hoc test was used to assess the difference; *p < 0.05; **p < 0.01; ***p < 0.001

Correct Fig. 3:

**Fig. 3:**
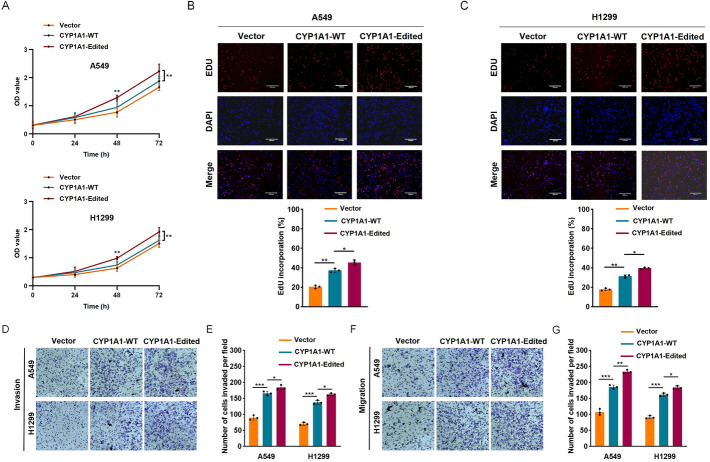
CYP1A1 A-to-I RNA editing confers enhanced tumorigenicity. **A** The growth rates of the indicated stable cell lines was measured by CCK-8. OD, optical density. EdU assays revealed that CYP1A1 A-to-I RNA editing enhanced the proliferation ability of indicated A549 (**B**) and H1299 (**C**) cells. Scale bars: 330 μm. The invasion (**D, E**) and migration abilities (**F, G**) of indicated stable cell lines were enhanced by CYP1A1 A-to-I RNA editing through Transwell assays. Scale bars: 220 μm. Scale bars: 220 μm. Standard deviation (SD) of three independent experiments. One-way ANOVA with Tukey’s test as post hoc test was used to assess the difference; *p < 0.05; **p < 0.01; ***p < 0.001
