# Supplementary figures and images for: A novel mechanism for A-to-I RNA-edited CYP1A1 in promoting cancer progression in NSCLC
Source: Cell Mol Biol Lett. 2025 Apr 2;30:40. doi: 10.1186/s11658-025-00718-6 (PMC11966828; doi:10.1186/s11658-025-00718-6)

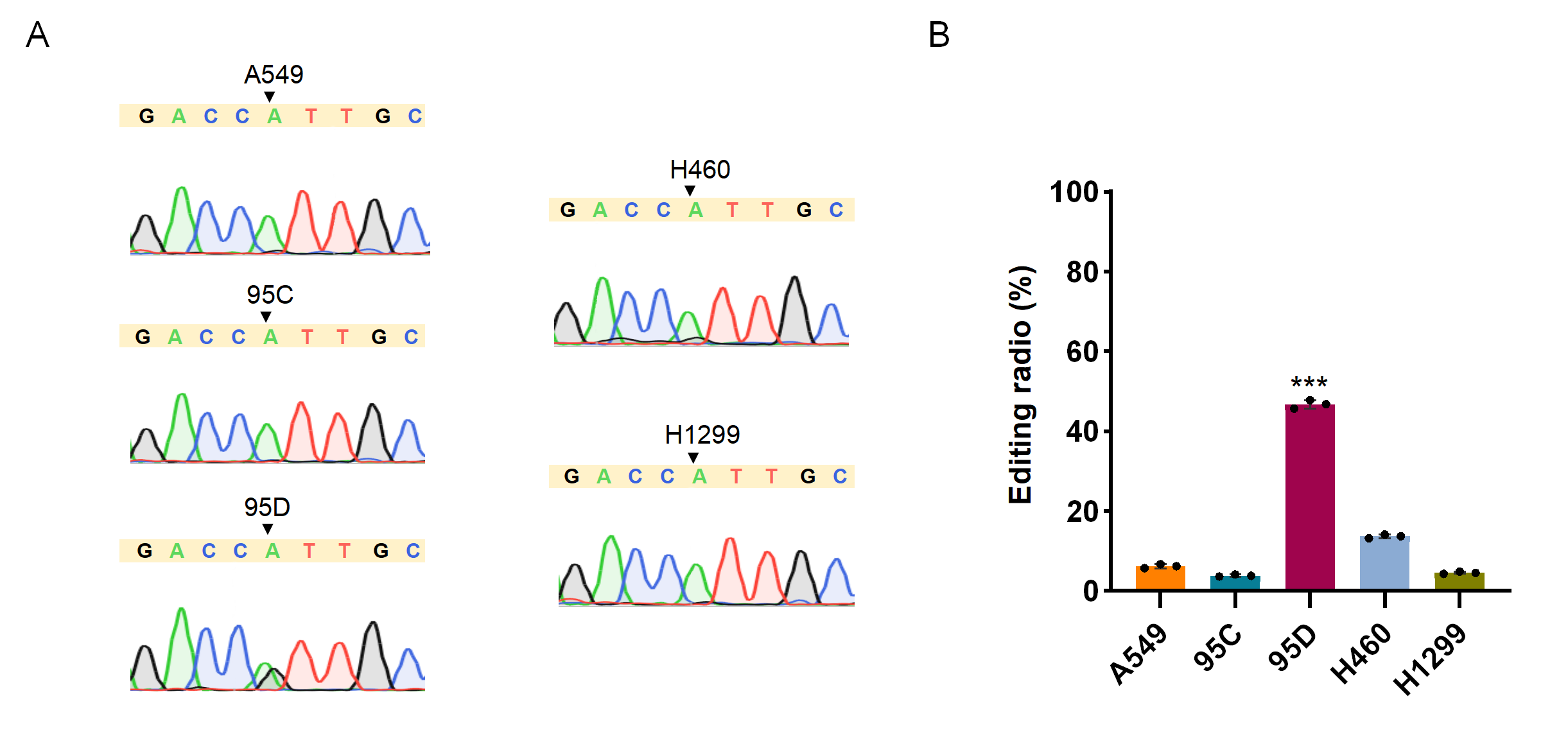

Supplement: Supplementary file 1 — Supplementary Material 1: Fig. S1. (A) Sequence analysis of CYP1A1 in edited regions from different lung cancer cell lines, such as A549, H1299, H460, 95C, and 95D. PCR fragments were obtained from cDNA (mRNA) using primers described in “Methods.” Samples were subjected to Sanger sequencing. (B) The CYP1A1 editing level of each cell line. Fig. S2. (A) Western blots of CYP1A1 overexpression in A549 and H1299 cells. Flag tag was used to quantify the CYP1A1 protein expression level, and GAPDH was used as loading control. (Control: control vector, WT: CYP1A1 wild-type, Edited: CYP1A1 edited-type). (B) Changes in CYP1A1 editing level after transfection with different types of CYP1A1 by Sanger sequencing. Three independent biological replicates were carried out in this experiment. One-way ANOVA with Tukey’s test as post hoc test was used to assess the difference; *** < 0.001. Fig. S3 .(A) Sequence chromatograms was detected in gDNA samples of A549 and A549I462V cells. (B) Assessment of the proliferation of A549 and A549I462V cells by EdU assays. (C) Wound healing of A549 and A549I462V cells. Scale bars, 550 μm. (D) The invasion and migration abilities of A549 and A549I462V cells. Scale bars, 210 μm. Standard deviation (SD) of three independent experiments. One-way ANOVA with Tukey’s test as post hoc test was used to assess the difference; **, p < 0.01; ***, p < 0.001. Fig. S4. (A, B) Images of HE and IHC staining by anti-PD-L1 antibody in orthotopic tumors (n = 5 per group). Scale bars: 50 μm. (C) H-score of Ki67 in orthotopic tumors, standard deviation (SD) of three independent experiments. One-way ANOVA with Tukey’s test as post hoc test was used to assess the difference. **, p < 0.01. [file 11658_2025_718_MOESM1_ESM.zip › Fig S1.tif]

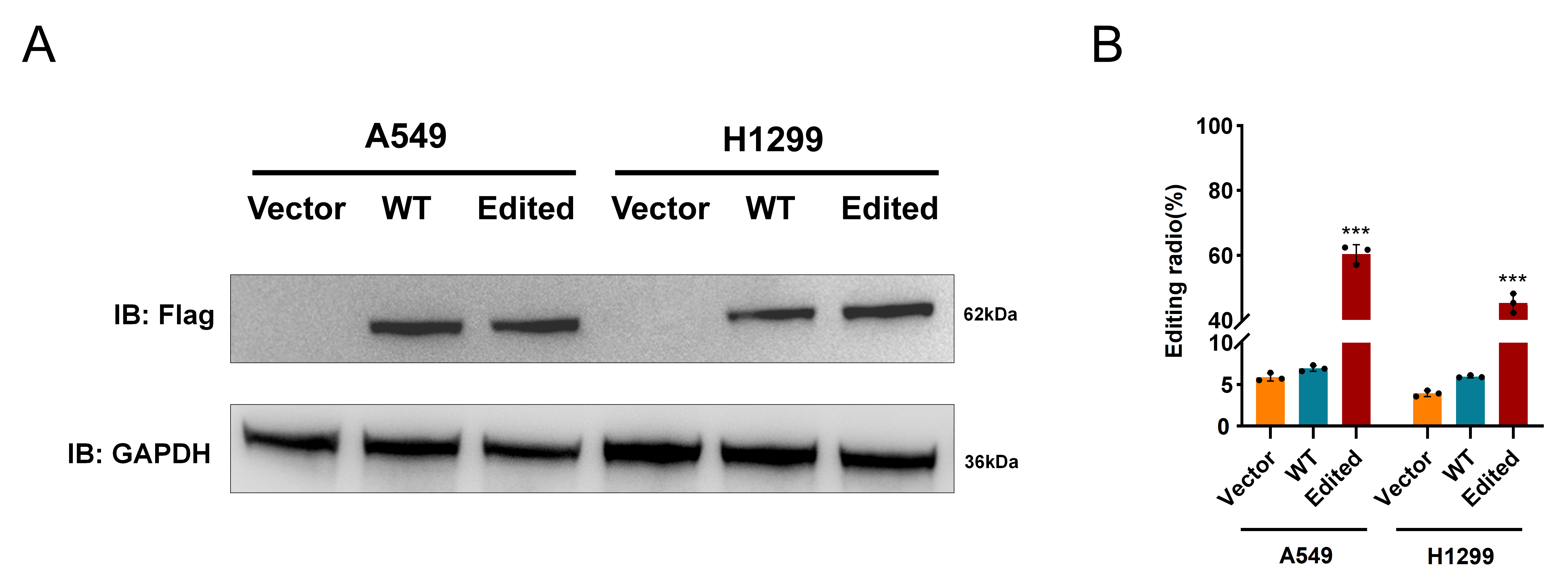

Supplement: Supplementary file 1 — Supplementary Material 1: Fig. S1. (A) Sequence analysis of CYP1A1 in edited regions from different lung cancer cell lines, such as A549, H1299, H460, 95C, and 95D. PCR fragments were obtained from cDNA (mRNA) using primers described in “Methods.” Samples were subjected to Sanger sequencing. (B) The CYP1A1 editing level of each cell line. Fig. S2. (A) Western blots of CYP1A1 overexpression in A549 and H1299 cells. Flag tag was used to quantify the CYP1A1 protein expression level, and GAPDH was used as loading control. (Control: control vector, WT: CYP1A1 wild-type, Edited: CYP1A1 edited-type). (B) Changes in CYP1A1 editing level after transfection with different types of CYP1A1 by Sanger sequencing. Three independent biological replicates were carried out in this experiment. One-way ANOVA with Tukey’s test as post hoc test was used to assess the difference; *** < 0.001. Fig. S3 .(A) Sequence chromatograms was detected in gDNA samples of A549 and A549I462V cells. (B) Assessment of the proliferation of A549 and A549I462V cells by EdU assays. (C) Wound healing of A549 and A549I462V cells. Scale bars, 550 μm. (D) The invasion and migration abilities of A549 and A549I462V cells. Scale bars, 210 μm. Standard deviation (SD) of three independent experiments. One-way ANOVA with Tukey’s test as post hoc test was used to assess the difference; **, p < 0.01; ***, p < 0.001. Fig. S4. (A, B) Images of HE and IHC staining by anti-PD-L1 antibody in orthotopic tumors (n = 5 per group). Scale bars: 50 μm. (C) H-score of Ki67 in orthotopic tumors, standard deviation (SD) of three independent experiments. One-way ANOVA with Tukey’s test as post hoc test was used to assess the difference. **, p < 0.01. [file 11658_2025_718_MOESM1_ESM.zip › Fig S2.tif]

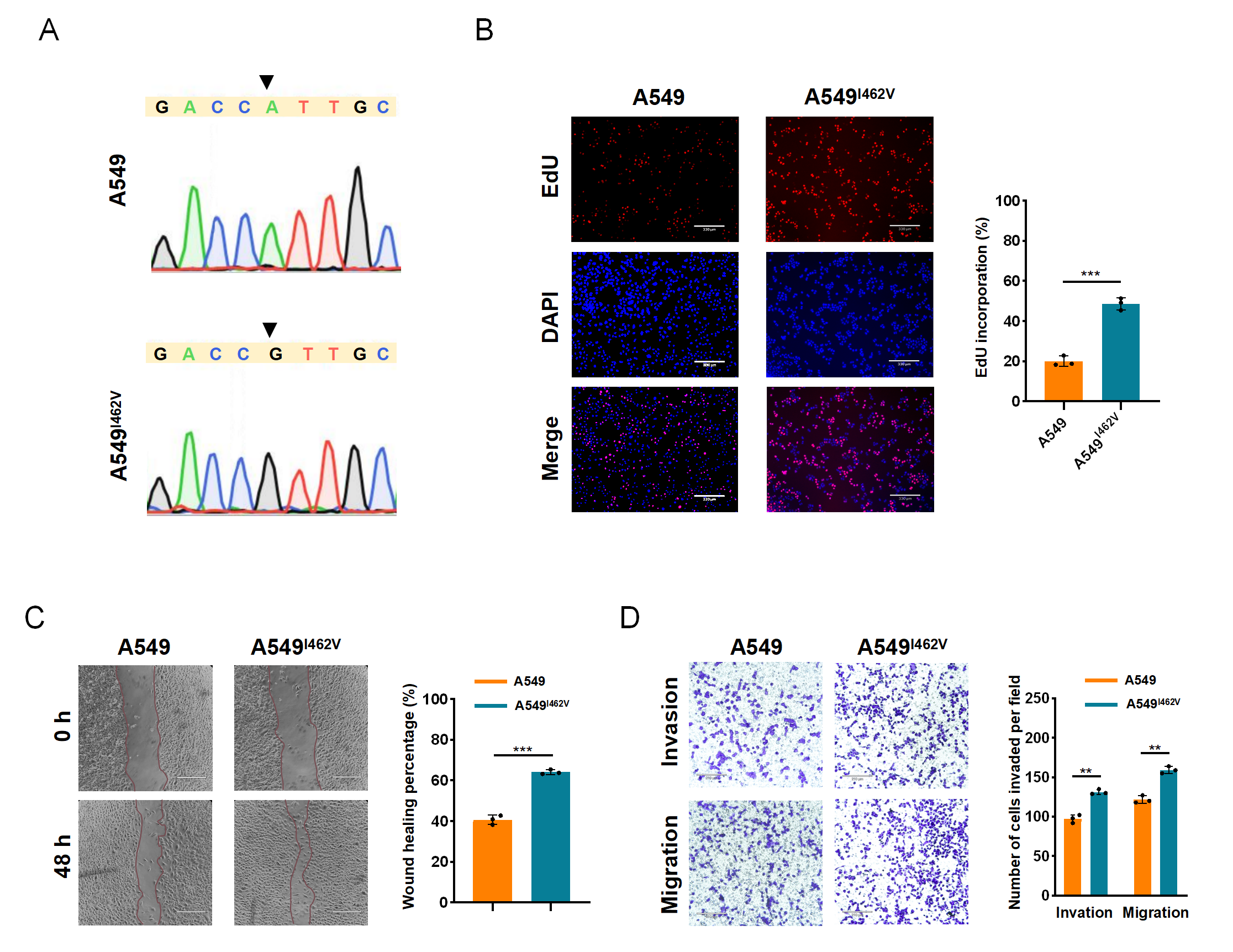

Supplement: Supplementary file 1 — Supplementary Material 1: Fig. S1. (A) Sequence analysis of CYP1A1 in edited regions from different lung cancer cell lines, such as A549, H1299, H460, 95C, and 95D. PCR fragments were obtained from cDNA (mRNA) using primers described in “Methods.” Samples were subjected to Sanger sequencing. (B) The CYP1A1 editing level of each cell line. Fig. S2. (A) Western blots of CYP1A1 overexpression in A549 and H1299 cells. Flag tag was used to quantify the CYP1A1 protein expression level, and GAPDH was used as loading control. (Control: control vector, WT: CYP1A1 wild-type, Edited: CYP1A1 edited-type). (B) Changes in CYP1A1 editing level after transfection with different types of CYP1A1 by Sanger sequencing. Three independent biological replicates were carried out in this experiment. One-way ANOVA with Tukey’s test as post hoc test was used to assess the difference; *** < 0.001. Fig. S3 .(A) Sequence chromatograms was detected in gDNA samples of A549 and A549I462V cells. (B) Assessment of the proliferation of A549 and A549I462V cells by EdU assays. (C) Wound healing of A549 and A549I462V cells. Scale bars, 550 μm. (D) The invasion and migration abilities of A549 and A549I462V cells. Scale bars, 210 μm. Standard deviation (SD) of three independent experiments. One-way ANOVA with Tukey’s test as post hoc test was used to assess the difference; **, p < 0.01; ***, p < 0.001. Fig. S4. (A, B) Images of HE and IHC staining by anti-PD-L1 antibody in orthotopic tumors (n = 5 per group). Scale bars: 50 μm. (C) H-score of Ki67 in orthotopic tumors, standard deviation (SD) of three independent experiments. One-way ANOVA with Tukey’s test as post hoc test was used to assess the difference. **, p < 0.01. [file 11658_2025_718_MOESM1_ESM.zip › Fig S3.tif]

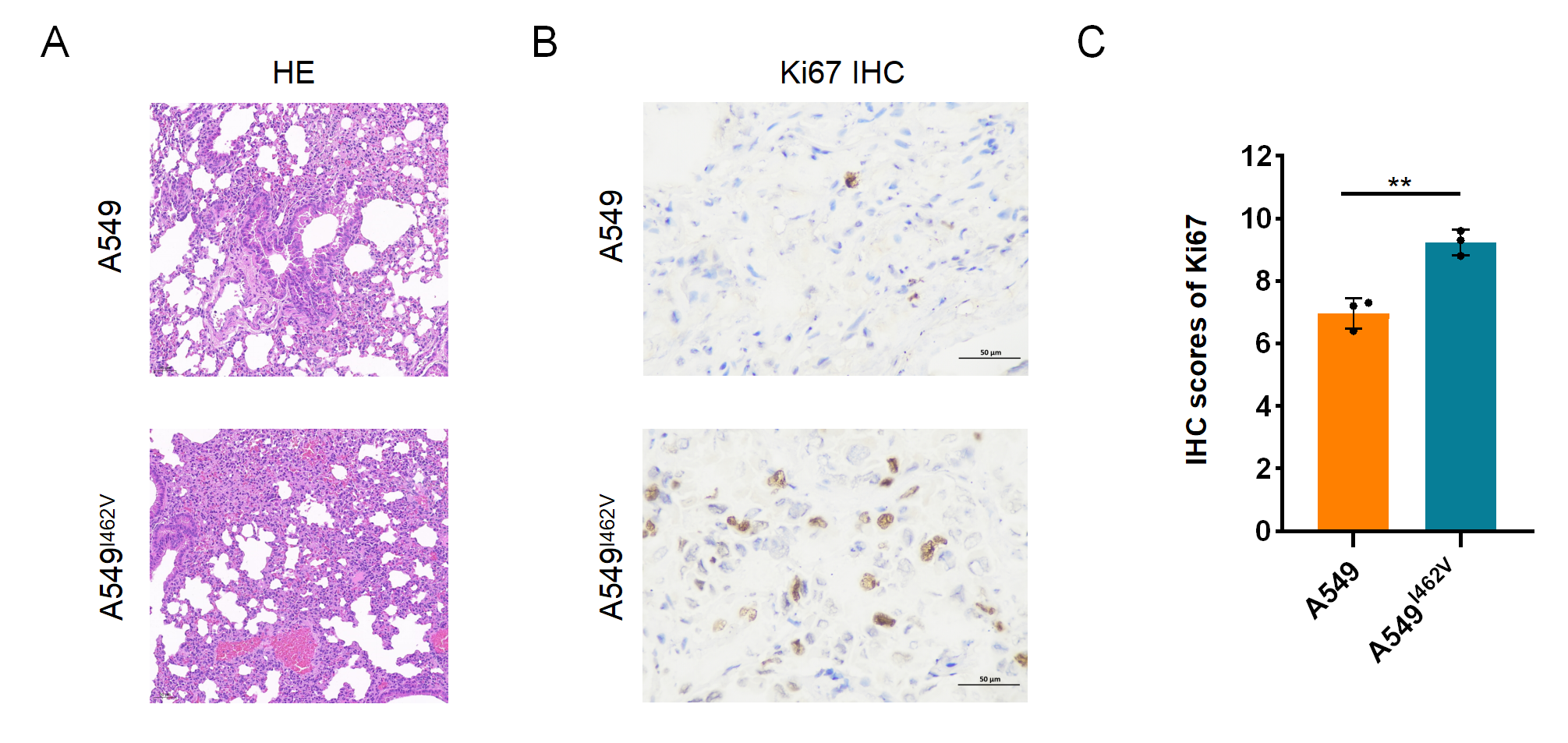

Supplement: Supplementary file 1 — Supplementary Material 1: Fig. S1. (A) Sequence analysis of CYP1A1 in edited regions from different lung cancer cell lines, such as A549, H1299, H460, 95C, and 95D. PCR fragments were obtained from cDNA (mRNA) using primers described in “Methods.” Samples were subjected to Sanger sequencing. (B) The CYP1A1 editing level of each cell line. Fig. S2. (A) Western blots of CYP1A1 overexpression in A549 and H1299 cells. Flag tag was used to quantify the CYP1A1 protein expression level, and GAPDH was used as loading control. (Control: control vector, WT: CYP1A1 wild-type, Edited: CYP1A1 edited-type). (B) Changes in CYP1A1 editing level after transfection with different types of CYP1A1 by Sanger sequencing. Three independent biological replicates were carried out in this experiment. One-way ANOVA with Tukey’s test as post hoc test was used to assess the difference; *** < 0.001. Fig. S3 .(A) Sequence chromatograms was detected in gDNA samples of A549 and A549I462V cells. (B) Assessment of the proliferation of A549 and A549I462V cells by EdU assays. (C) Wound healing of A549 and A549I462V cells. Scale bars, 550 μm. (D) The invasion and migration abilities of A549 and A549I462V cells. Scale bars, 210 μm. Standard deviation (SD) of three independent experiments. One-way ANOVA with Tukey’s test as post hoc test was used to assess the difference; **, p < 0.01; ***, p < 0.001. Fig. S4. (A, B) Images of HE and IHC staining by anti-PD-L1 antibody in orthotopic tumors (n = 5 per group). Scale bars: 50 μm. (C) H-score of Ki67 in orthotopic tumors, standard deviation (SD) of three independent experiments. One-way ANOVA with Tukey’s test as post hoc test was used to assess the difference. **, p < 0.01. [file 11658_2025_718_MOESM1_ESM.zip › Fig S4.tif]
