# Supplementary material for: A novel mechanism for A-to-I RNA-edited CYP1A1 in promoting cancer progression in NSCLC
Source: Cell Mol Biol Lett. 2025 Apr 2;30:40. doi: 10.1186/s11658-025-00718-6 (PMC11966828; doi:10.1186/s11658-025-00718-6)
Supplement: Supplementary file 2 — Supplementary Material 2. [file 11658_2025_718_MOESM2_ESM.docx]

**Table S1. Summary of clinical features and A-to-I (G) RNA editing levels of 103 NSCLC serum cases.**

| Case | Gender | Age | Stage | Editing level (%) |
| --- | --- | --- | --- | --- |
| 2 | M | 61 | II | 42.1 |
| 4 | M | 38 | I | 31.3 |
| 12 | F | 29 | I | 17.2 |
| 15 | M | 64 | I | 53.3 |
| 16 | F | 71 | II | 45.9 |
| 21 | M | 48 | II | 57.8 |
| 25 | M | 53 | III | 62.6 |
| 27 | F | 71 | III | 58.8 |
| 28 | M | 50 | IV | 25.9 |
| 30 | M | 41 | IV | 58.5 |
| 33 | M | 55 | III | 37.8 |
| 39 | M | 67 | II | 37.2 |
| 45 | M | 26 | II | 58.7 |
| 47 | M | 46 | II | 46.6 |
| 55 | F | 60 | I | 18.4 |
| 59 | M | 66 | III | 58.2 |
| 60 | M | 69 | III | 85.6 |
| 62 | M | 72 | IV | 96.9 |
| 66 | F | 75 | IV | 58.4 |
| 69 | M | 57 | III | 39.2 |
| 71 | M | 37 | II | 47.3 |
| 76 | M | 49 | III | 28.9 |
| 85 | F | 52 | IV | 57.3 |
| 86 | M | 63 | II | 58.1 |
| 89 | M | 42 | IV | 90.1 |
| 90 | M | 68 | IV | 22.3 |
| 96 | M | 38 | II | 57.9 |
| 103 | F | 61 | III | 57.5 |
| 107 | F | 70 | IV | 73.2 |
| 116 | M | 68 | II | 62.8 |
| 119 | M | 67 | II | 49.9 |
| 127 | M | 63 | I | 31.7 |
| 134 | M | 24 | I | 19.7 |
| 135 | M | 36 | III | 55.2 |
| 138 | F | 62 | I | 63.5 |
| 144 | M | 78 | III | 52.9 |
| 149 | F | 63 | IV | 58.6 |
| 156 | F | 66 | III | 82.5 |
| 158 | M | 65 | II | 43.8 |
| 160 | M | 72 | IV | 79.4 |
| 163 | M | 39 | II | 51.6 |
| 166 | M | 43 | III | 73.1 |
| 172 | M | 47 | III | 29.1 |
| 177 | F | 69 | IV | 59.6 |
| 184 | M | 34 | II | 44.7 |
| 192 | M | 42 | IV | 60.3 |
| 196 | M | 33 | III | 29.7 |
| 205 | M | 69 | III | 71.9 |
| 211 | M | 52 | II | 16.9 |
| 216 | M | 60 | IV | 71.3 |
| 218 | M | 57 | I | 33.8 |
| 223 | F | 72 | IV | 40.9 |
| 229 | M | 54 | IV | 78.8 |
| 237 | M | 49 | III | 40.5 |
| 240 | F | 51 | III | 60.8 |
| 245 | M | 38 | I | 18.8 |
| 247 | M | 62 | IV | 90.7 |
| 251 | F | 55 | I | 54.9 |
| 252 | F | 77 | IV | 15.3 |
| 253 | M | 71 | I | 33.4 |
| 259 | M | 72 | IV | 52.8 |
| 266 | M | 66 | III | 61.4 |
| 267 | M | 45 | I | 21.5 |
| 277 | M | 66 | II | 75.9 |
| 281 | M | 64 | III | 41.6 |
| 288 | M | 43 | IV | 49.3 |
| 289 | M | 68 | III | 51.7 |
| 295 | F | 43 | IV | 48.9 |
| 297 | M | 65 | II | 59.2 |
| 306 | M | 71 | IV | 92.6 |
| 314 | M | 66 | III | 48.4 |
| 316 | F | 69 | III | 58.8 |
| 319 | M | 51 | III | 78.2 |
| 325 | M | 64 | IV | 42.4 |
| 326 | M | 82 | IV | 61.8 |
| 328 | F | 76 | III | 94.8 |
| 334 | M | 62 | II | 58.9 |
| 346 | M | 37 | II | 74.8 |
| 349 | M | 50 | III | 44.8 |
| 350 | M | 67 | IV | 69.2 |
| 358 | F | 79 | IV | 53.3 |
| 364 | M | 83 | III | 47.8 |
| 368 | M | 66 | III | 64.1 |
| 377 | F | 55 | II | 55.3 |
| 382 | F | 72 | III | 43.6 |
| 389 | M | 80 | IV | 96.2 |
| 390 | M | 79 | III | 62.4 |
| 395 | F | 74 | II | 73.9 |
| 402 | M | 31 | I | 23.1 |
| 406 | M | 43 | IV | 54.6 |
| 417 | M | 62 | IV | 67.4 |
| 422 | F | 53 | II | 64.9 |
| 426 | M | 39 | I | 25.4 |
| 441 | M | 77 | III | 54.6 |
| 447 | M | 80 | IV | 47.2 |
| 453 | M | 79 | III | 45.5 |
| 458 | F | 58 | IV | 66.9 |
| 466 | M | 63 | I | 35.5 |
| 471 | F | 72 | I | 65.8 |
| 475 | M | 61 | III | 77.1 |
| 483 | F | 67 | II | 56.8 |
| 491 | M | 68 | III | 53.3 |
| 493 | M | 62 | III | 67.8 |

**Table S2. Correlation between overediting of *CYP1A1* with clinicopathological**

**features in 34 NSCLC patients.**

| Clinical features | Number | *CYP1A1* editing level | | *p*-value |
| --- | --- | --- | --- | --- |
|  |  | Overediting(-) | Overediting(+)^a^ |  |
| Age (years) |  |  |  | 0.278 |
| >60 | 23 | 7 (53.8%) | 16 (76.2%) |  |
| ≤60 | 11 | 6 (46.2%) | 5 (23.8%) |  |
| Gender |  |  |  | 0.191 |
| Male | 25 | 8 (61.5%) | 17 (80.9%) |  |
| Female | 9 | 5 (38.5%) | 4 (19.1%) |  |
| Tumor stage |  |  |  | 0.483 |
| Ⅰ/Ⅱ | 29 | 14 (87.5%) | 15 (83.3%) |  |
| Ⅲ | 5 | 2 (12.5%) | 3 (16.7%) |  |
| Smoking history |  |  |  | 0.117 |
| Smoker | 19 | 9 (52.9%) | 10 (58.8%) |  |
| Non-smoker | 15 | 8 (47.1%) | 7 (41.2%) |  |
| Recurrence or Metastasis |  |  |  | <0.001 |
| Absent | 19 | 13 (76.5%) | 6 (35.2%) |  |
| Present | 15 | 4 (23.5%) | 11 (64.8%) |  |

^a^ NSCLC specimens demonstrating an increase of not less than 10% editing level than matched NAT samples are classified as“Overediting (+)” Group

**Table S3. The sh-RNAs or sgRNA used in this study**

| sh-RNAs or sgRNA | Sequence(5’-3’) |
| --- | --- |
| sh-NC | TTCTCCGAACGTGTCACGT |
| sh-ADAR1#1 | CCTGTGGAATCCAGTGACATTGTGCCTAC |
| sh-ADAR1#2 | AGACTCCGTACCATGTCCTGTAGTGACAA |
| sh-ADAR2#1 | GCAGCTGAACGAGATCAAACC |
| sh-ADAR2#2 | GCATCAACGGCGAATACATGA |
| A549^I462V^-sgRNA | AAAGACCTCCCAGCGGGCAA |

**Table S4. Primes used in qRT-PCR and PCR analysis in this study**

| Primers | Sequence(5’-3’) |
| --- | --- |
| CYP1A1 | F: TGAGTTCCTACCTGAACGGTTT |
|  | R: ATGGGGGTCATGTCCACCTTCA |
| GAPDH-qRT | F: CAGGAGGCATTGCTGATGAT |
|  | R: GAAGGCTGGGGCTCATTT |
| ADAR1-qRT | F: CCCTTCAGCCACATCCTTC |
|  | R: GCCATCTGCTTTGCCACTT |
| ADAR2-qRT | F: CTGACACGCTCTTCAATGGTT |
|  | R: GGCGCAGTTCGTTCAAGAT |
| CYP1A1-WT | F: CGGGATCCATGCTTTTCCCAATCTCC(Ba) |
|  | R: GCTCTAGAAGAGCGCAGCTGCATTTG(Xb) |
| CYP1A1-Edited | F: AAGTGTATCGGTGAGACCATTGCCCGCTGG |
|  | R: AGACCTCCCAGCGGGCAAGGGTCTCACCGA |
| ADAR1-OE | F: GGGGTACCCACCGAAAGAGGCAGGAACAC(Kp) |
|  | R: GCTCTAGACTATACTGGGCAGAGATAAAAGT(Xb) |
| ADAR2-OE | F: GGGGTACCCACCATGGATATAGAAGATGA(Kp) |
|  | R: CCCTCGAGTCAGGGCGTGAGTGAGAACT(Xh) |
| PCAGGS-HA-HO-1 | F: CGGAATTCATGGAGCGTCCGCAACCC(Ec) |
|  | R: CCCTCGAGTCACATGGCATAAAGCCC(Xh) |
| pCMV-C-Flag-CYP1A1 | F: CGGGATCCATGCTTTTCCCAATCTCC(Ba) |
|  | R: CCCTCGAGAGAGCGCAGCTGCATTTG(Xh) |

^a^ Underscored sequences indicate introduced restriction sites. Ba, *Bam*HI; Ec, *Eco*RI; Kp, *Kpn*I; Xb, *Xba*I; and Xh, *Xho*I. The nucleotides in red font indicate sites where point mutations were introduced.
